# Visible-Light Actinometry and Intermittent Illumination as Convenient Tools to Study Ru(bpy)_3_Cl_2_ Mediated Photoredox Transformations

**DOI:** 10.1038/srep16397

**Published:** 2015-11-18

**Authors:** Spencer P. Pitre, Christopher D. McTiernan, Wyatt Vine, Rebecca DiPucchio, Michel Grenier, Juan C. Scaiano

**Affiliations:** 1 Department of Chemistry and Biomolecular Sciences and Centre for Catalysis Research and Innovation, University of Ottawa, 10 Marie Curie, Ottawa, Ontario, K1N 6N5, Canada

## Abstract

Photoredox catalysis provides many green opportunities for radical-mediated synthetic transformations. However, the determination of the underlying mechanisms has been challenging due to lack of quantitative methods that can be easily implemented in synthetic labs, where this research tends to be centered. We report here on the development, characterization and calibration of a novel actinometer based on the photocatalyst tris(2,2′-bipyridyl)ruthenium(II) chloride (Ru(bpy)_3_Cl_2_). By using the same molecule as the photocatalyst and the actinometer, we eliminate problems associated with matching sample spectral distribution, lamp-sample spectral overlap and other problems intrinsic to doing quantitative photochemistry in a laboratory that has little expertise in this area. In order to validate our actinometer system in determining the quantum yield of a Ru(bpy)_3_Cl_2_ photosensitized reaction, we test the Ru(bpy)_3_Cl_2_ catalyzed oxidation of benzhydrol to benzophenone as a model chain reaction. We also revive the rotating sector method by updating the technique for modern LED technologies and demonstrate how intermittent illumination on the timescale of milliseconds to seconds can help probe a chain reaction, using the benzhydrol to benzophenone oxidation to validate the technique. We envision these methods to have great implications in the field of photoredox catalysis, providing researchers with valuable research tools.

Over the last decade, the field of photoredox catalysis has gained increasing attention due to its wide applicability in sustainable organic synthesis^1^. One of the key driving forces in the growth of this field relies on the use of photosensitizers that take advantage of “visible-light” wavelengths (400–700 nm) of the electromagnetic spectrum, providing milder conditions than traditional UV-promoted photochemical reactions. This is enticing for chemists looking for “greener” reaction conditions, however, perhaps more importantly, it also aids in avoiding possible side reactions or product decomposition, as many simple organic molecules do not absorb in the visible range. A large range of photosensitizers, including transition-metal complexes^2^ and organic photosensitizers^3^, have been employed in a wide variety of chemical transformations. However, the vast majority of these transformations have been catalyzed by tris(2,2′-bipyridyl) ruthenium(II) chloride (Ru(bpy)_3_Cl_2_, see Fig. 1). Ru(bpy)_3_Cl_2_, despite being a precious metal catalyst, offers a variety of advantages, which include a strong visible-light absorption and a stable, long-lived excited state lifetime^4^. Like all diamagnetic molecules, upon excitation to the lowest energy excited-state, Ru(bpy)_3_Cl_2_ becomes both a stronger electron donor and acceptor than in the ground state^5^. Coupled with its long-lived triplet metal-to-ligand charge transfer (^3^MLCT) excited state, this allows for favourable conditions for single-electron-transfer (SET) reactions, which has been widely utilized in the recent photocatalysis literature.

Despite all the recent advancements in this field, many of the new discoveries take place in the absence of knowledge about excited-state kinetics as well as an understanding of the underlying mechanisms. Recently, our group, along with others, have demonstrated the usefulness of laser flash photolysis techniques for determining bimolecular rate constants of mechanistically key steps, which provided insights into the overall reaction mechanism^6^,^7^,^8^,^9^,^10^. Other techniques that could be powerful tools would be visible-light actinometry along with the “rotating sector” (RS) method^11^,^12^. In many cases throughout the literature, proposed mechanisms often suggest the possibility of a chain-propagating reaction, and are typically probed through intermittent irradiation with on/off times in the minute timescale. This method, however, does not provide any insights into a possible chain reaction, as chain reactions are normally terminated within milliseconds-to-seconds after the illumination source is turned off. One method of probing a chain-propagating mechanism is to determine the quantum yield of the reaction; however this is typically not investigated. This reflects that synthetic chemistry laboratories lack access to the necessary instrumentation, or chemical actinometers, to perform these studies. In order to overcome this problem, we envisioned that a visible-light actinometer based on Ru(bpy)_3_Cl_2_ in combination with well-known singlet oxygen chemistry could be the perfect solution^13^. With this system, one could perform a reaction using Ru(bpy)_3_Cl_2_ as the photocatalyst with the same concentration of Ru(bpy)_3_Cl_2_ as the actinometer, and the quantum yield could easily be determined, as the delicate matching of light sources, absorption profiles and efficiency of light absorption becomes trivial given that the photocatalyst and actinometer are the same molecule.

However, in many cases one cannot simply rely on actinometry alone to determine whether or not a propagating chain is involved in the reaction. For example, having a quantum yield (Φ) < 1 does not imply that no chain reaction is involved. If the initiation step is inefficient, this could result in a lower value for the 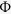, even if chain propagation is involved. On the other hand, a Φ > 1 also does not definitively identify the involvement of a chain mechanism. For example, one could envision a reaction involving a homolytic cleavage of a symmetrical molecule as easily having a quantum yield as high as 2 if a single cleaving event results in two product molecules; in other words, stoichiometric factors can result in values in the 1-to-2 range. Therefore having both a convenient and reliable visible-light actinometer based on Ru(bpy)_3_Cl_2_ and modernized RS would not only allow determination of the Φ for a reaction, but could also enable the measurement of chain propagation lifetimes^12^,^14^,^15^,^16^,^17^.

We report here the development, characterization and calibration of a visible-light actinometer based on the ubiquitous photocatalyst, Ru(bpy)_3_Cl_2_, as well as a revised RS method based on a pulsed LED system to probe possible chain reactions in photoredox catalysis. Lastly, we validate the utility of these tools by characterizing the Ru(bpy)_3_Cl_2_ catalyzed oxidation of benzhydrol to benzophenone using 4-cyano-*N*-methoxypyridinium as the chain amplifier.

## Results

### Calibration of a Ru(bpy)_3_Cl_2_ Based Actinometer

Determining the Φ of a photochemical reaction requires the ability to measure the number of molecules consumed or produced during a given period of irradiation as well as the number of photons absorbed by the system during the same period. It is important to distinguish here between incident and absorbed photons, as only photons absorbed by the sample can produce a chemical change. For this reason, the Φ of a photochemical reaction can be defined by equation (1).


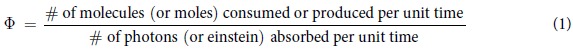


Considering that in most cases one can easily determine the number of moles consumed or produced through a variety of different analytical techniques, all that is required to determine the quantum yield of a reaction is the einsteins (i.e., moles of photons) absorbed by the sample during the irradiation. The standard approach is to perform actinometry experiments, where one can determine the energy delivered to a particular sample within a defined spectral range and geometry^18^. Although any photoactive system for which the quantum yield is known could be used as an actinometer, the ability to quickly and conveniently determine the number of actinometer molecules reacted can really influence the utility of the system. For example, one of the most widely employed chemical actinometers for UV and visible-light wavelengths up to 500 nm is the ferrioxalate actinometer, in which the number of molecules reacted can easily be determined through UV-Vis spectrophotometry^19^. However, there exist some drawbacks to this system. Firstly, the potassium ferrioxalate must be synthesized, and secondly a buffered solution of phenanthroline must be added as a developing agent. Further spectral matching taking into account the emission spectrum of the light source and the absorption spectrum of the sample can be challenging and require familiarity with actinometric methods. Thus, we decided to calibrate our Ru(bpy)_3_Cl_2_ actinometer using the ferrioxalate actinometer due to the reproducible reliability of the ferrioxalate system under laboratory conditions where the tools to match the photon absorbed by actinometer and sample are readily available.

**Figure 2:**
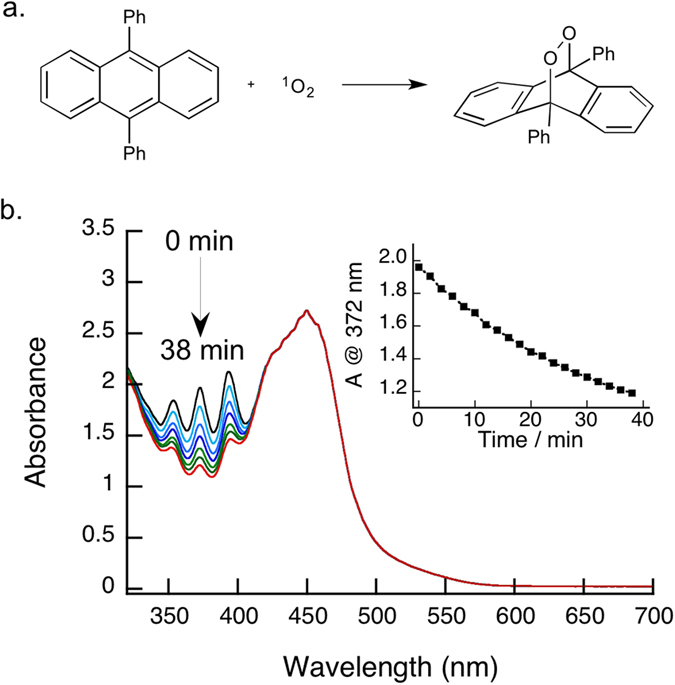
Oxidation of DPA and Actinometric Analysis. (**a**) The reaction of 1,9-diphenylanthracene (DPA) with ^1^O_2_ yielding its corresponding endoperoxide. (**b**) Absorption spectra of a typical actinometry experiment performed with Ru(bpy)_3_Cl_2_ (0.19 mM) and DPA (0.10 mM) in acetonitrile and irradiated with a 460 nm LED equipped with a 440 nm notch filter. Inset: Absorption at 372 nm vs. irradiation time corresponding to data from (**b**).

For our Ru(bpy)_3_Cl_2_ actinometer, we chose to base it on well-known singlet oxygen chemistry, employing the oxidation of 1,9-diphenylanthracene (DPA) to its corresponding endoperoxide (see Fig. 2a). The consumption of DPA can be easily monitored through UV-Vis spectrophotometry, as DPA exhibits a distinct absorption line at 372 nm, allowing for easy quantification of the reaction. Importantly, this peak is not obscured by the broad absorption band from the ^3^MLCT of Ru(bpy)_3_Cl_2_ (λ_max_ = 454 nm) and any overlap can be easily corrected, allowing for easy quantification in the presence of the photosensitizer (see Fig. 2b). Furthermore, employing DPA in our visible-light actinometer system comes with another added advantage in that DPA does not absorb in the visible-light region (see Figure S1 of SI). Since Ru(bpy)_3_Cl_2_ is typically excited with visible-light in photoredox processes, possible side reactions from the direct excitation of DPA are not an issue.

A typical experiment with our Ru(bpy)_3_Cl_2_ actinometer system is shown in Fig. 2b. As demonstrated, the consumption of DPA through its oxidation by ^1^O_2_ generated from Ru(bpy)_3_Cl_2_ can be easily monitored at 372 nm. By using the extinction coefficient (ε) at 372 nm, which we measured to be 11,100 M^−1^ cm^−1^ (see Figure S2 of SI), the concentration of DPA remaining in the sample can be determined at any given time using the Beer-Lambert law, which allows us to determine the conversion of DPA to its corresponding endoperoxide in a given irradiation time.

In order to determine the Φ of our actinometer system, we must first determine the intensity of our light source. In order to determine this value, an actinometer with a known quantum yield at the same wavelength as our light source needed to be chosen. As previously mentioned, we chose the ferrioxalate system due to its known reliability in the wavelength range we required. Both the absorbance of the sample and actinometer were matched at 440 nm and a notch filter at this wavelength was used to simplify lamp-sample spectral overlap. We note that this filter was used only to calibrate the Ru(bpy)_3_Cl_2_ actinometer, and is not needed by users employing the Ru(bpy)_3_Cl_2_ actinometer itself. The only requirement is that the light source has no significant emission below 400 nm to avoid direct excitation of DPA.

By monitoring the absorbance of the ferrioxalate complex at 510 nm with irradiated and non-irradiated samples, we were able to calculate the incident intensity of our light source to be 6.5 × 10^−9^ einsteins s^−1^ (see Section F in SI for calculation). Finally, by employing the following equation:


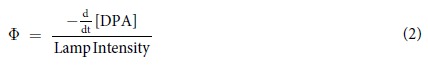


we can calculate the Φ of our Ru(bpy)_3_Cl_2_ visible-light actinometer system to be 0.019 ± 0.001 (excluding error intrinsic to the ferrioxalate actinometer). While the Φ of our actinometer is considerably lower than other visible-light systems, such as the ferrioxalate system, it provides the advantage of being easier to work with compared to the other chemical systems, as our system can withstand the longer irradiation times and higher light intensities typically employed in photoredox transformations. Further, no developing agent is required, as is the case in the ferrioxalate system. Our system is also comprised of all commercially available reagents, shows a linear relationship to the power dependence of the light source (see Figure S4 of SI), and there is no concerns relating to wavelength specificity, as the actinometer is also the photocatalyst.

**Figure 3:**
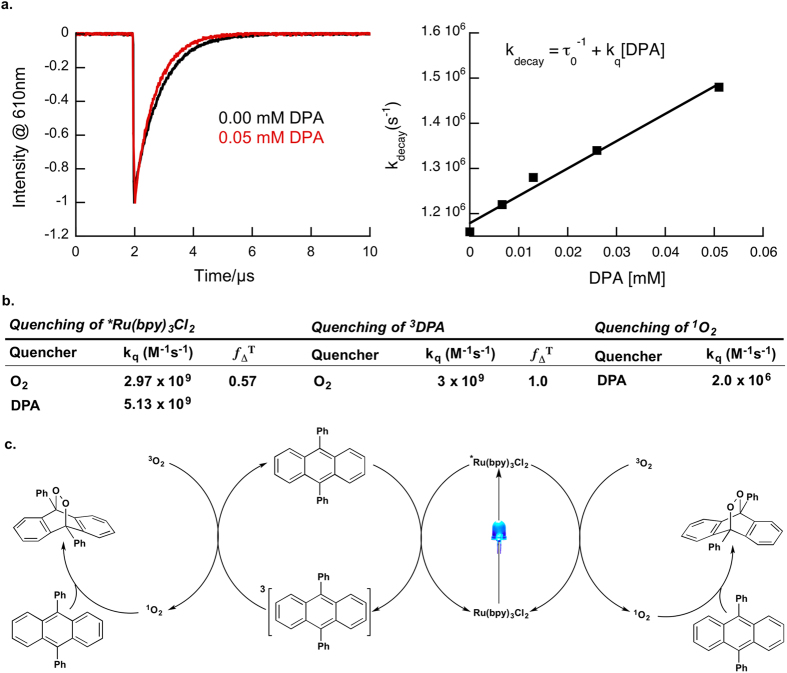
Mechanistic Investigation of the Ru(bpy)_3_Cl_2_ Actinometric System. (**a**) Laser flash photolysis data from a typical bimolecular quenching experiment for Ru(bpy)_3_Cl_2_ and DPA. (**b**) Bimolecular rate constants (k_q_) and ^1^O_2_ generation efficiency (*f*_Δ_^T^) of all the mechanistally key steps in our actinometer system^6^,^22^. (**c**) Generalized reaction scheme for the Ru(bpy)_3_Cl_2_ based actinometer. Note that the extreme left and right reactions are the same, with singlet oxygen being produced from different sensitization steps.

Due to the low efficiency of our Ru(bpy)_3_Cl_2_ actinometer, we decided to examine the kinetics of the system through laser flash photolysis techniques to help shed light behind the low Φ (see Fig. 3). Upon determining the bimolecular quenching constants for the key steps in the mechanism, equations (3) and (4) can be used to calculate the percentage of ^3^MLCTs quenched by oxygen and the percentage of ^1^O_2_ quenched by DPA under initial reaction conditions.


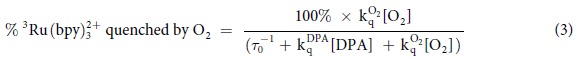



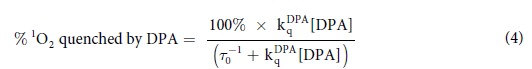


From the bimolecular quenching constants in Fig. 3, we were able to calculate using equation (3) that under initial reaction conditions, 77% of the ^3^MLCTs are intercepted by oxygen, while only 1.5% of the ^1^O_2_ produced is intercepted by DPA (equation (4)). Although it could be concluded that the low Φ obtained for our Ru(bpy)_3_Cl_2_ actinometer stems from the low quenching efficiency of ^1^O_2_ by DPA, given the quenching efficiencies above the Φ of our system is too large, even without considering the fact that only 57% of ^3^Ru(bpy)_3_Cl_2_ quenching events lead to ^1^O_2_ generation^20^. A possible explanation for the higher than expected quantum yield is that ^*^Ru(bpy)_3_Cl_2_ is acting as a triplet sensitizer, producing ^3^DPA which can in turn sensitize the production of ^1^O_2_ (see Section I of SI). Using equation (5), we calculate that 8.3% of the ^3^MLCTs are intercepted by DPA. Considering the long triplet lifetime of DPA (approx. 3 ms)^21^ and that we estimate ^3^DPA quenches O_2_ at a rate of 3 × 10^9^ M^−1^s^−1^,^22^ applying equation (6) we find that 99.9% of ^3^DPA is quenched by O_2_. Combining this with the fact that the efficiency for ^1^O_2_ generation (*f*_Δ_^T^) by ^3^DPA is 100%,^22^,^23^ it becomes clear that this secondary route to ^1^O_2_ can account for the higher than expected Φ. It may also be important to consider that the generation of ^1^O_2_ and its subsequent reaction involve the same reactants in the secondary pathway, although this does not eliminate the diffusional requirements as the original pair are both triplets, it may reduce them.


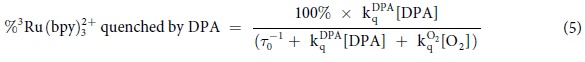



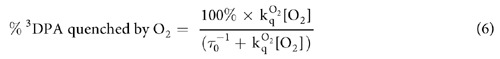


Importantly, the Φ for our actinometer is ultimately an experimental value and the proceeding discussion simply satisfies our curiosity and does not affect the value of 0.019 given above.

### Intermittent illumination as a chain diagnostics strategy: Revised RS method

While the RS method may not be as popular as it once was, it remains evident that many in the field of photoredox catalysis understand that one can probe a photo-initiated chain reaction through the use of intermittent illumination. However, as the lifetime of most chains are in the sub-second timescale, current attempts to establish whether or not a photoredox transformation involves a chain by testing the effect of switching the light source on and off on the time scale of minutes are futile. As might be expected the more appropriate test for a chain reaction is conceptually the RS method, where microsecond to second light pulses are employed.

Using existing LED technologies we envisioned the development of a new age RS method in which the RS would be replaced with a pulsed LED and that no moving parts would be involved. The LED would be powered by a constant current driver and controlled by a digital delay/pulse generator. Additional advantages of this system include a smaller physical footprint, digital control over the pulse length, light intensity, and light-to-dark ratio making the system quite versatile and user friendly. Note that in the traditional RS the light-to-dark ratio is fixed as it is determined by the configuration of open sectors in the rotating wheel. For further details on the design of the apparatus, refer to the SI.

Though the RS experiment was and is designed to be quantitative, we envision it as being used as more of a qualitative or semi-quantitative tool. For example, using the modified RS experiment one could qualitatively define a reaction as involving a chain by simply demonstrating a non-linear dependence between conversion and the rate of sample illumination, as one would expect that the rate of photon delivery should have no effect on a system free of chain reactions. However, the RS method remains to be one of the easiest ways to experimentally determine the average lifetime of a propagating chain (τ_s_)^24^.

The τ_s_ of a chain under steady state conditions is defined by equation (7).


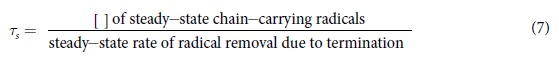


The problem with this is that under most conditions, the rate of the reaction is being measured under non-steady state conditions. Even though one of the main advantages of a light induced radical reaction is the ability to instantaneously initiate and interrupt radical formation by simply turning on or off the light source, there still remains a period of time between when the chain is initiated and realisation of the steady state, as well as another period of time between when the light is removed and the depletion of chain-carriers through termination reactions. Ideally one would determine τ_s_ by measuring the rate of reaction over a given time after which the illumination has been terminated, however as τ_s_ tends to be on the millisecond timescale and the amount of conversion during this time period would be minimal, it is difficult to accurately measure this value. Performing the RS experiment makes this measurement viable as one can average the result over multiple on-off cycles. For more information regarding the RS experiments see Section K of the supplementary data.

### Validation of new actinometry and RS methods: Characterization of the Oxidation of Benzhydrol

**Figure 4:**
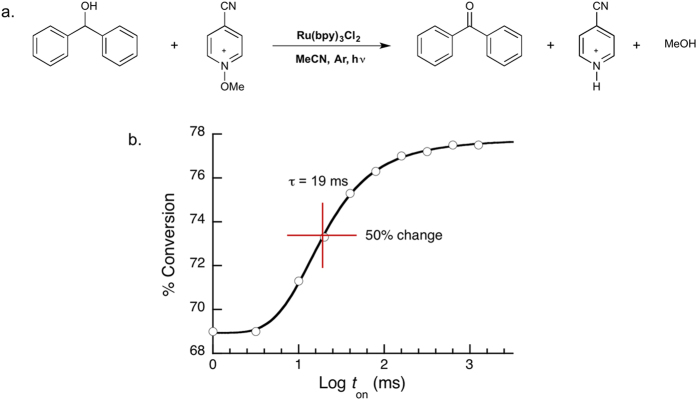
Characterization of the Photocatalyzed Oxidation of Benzhydrol. (**a**) Reaction scheme for the Ru(bpy)_3_Cl_2_ catalyzed oxidation of benzhydrol in the presence of 4-cyano-*N-*methoxypyridinium salt. (**b**) Conversion of benzhydrol to benzophenone as a function of Log (light on period in milliseconds [*t*_on_]). Reaction Conditions: Ru(bpy)_3_Cl_2_ (6 mM), benzhydrol (30 mM), and 4-cyano-*N-*methoxypyridinium tetrafluoroborate (30 mM) in acetonitrile (3 mL) in a quartz cuvette were degassed with argon and irradiated with a 460 nm LED.

In order to validate the usefulness of both the Ru(bpy)_3_Cl_2_ based actinometer and the intermittent illumination experiments, it was important to use these techniques to characterize a known chain reaction. It has been previously shown that in the presence of *N*-alkoxypyridium salts the photocatalyzed oxidation of benzhydrol to benzophenone becomes a chain reaction^25^. We thus decided that the oxidation of benzhydrol catalyzed by Ru(bpy)_3_Cl_2_ in the prescence of 4-cyano-*N-*methoxypyridinium tetrafluoroborate would be an ideal test reaction for the new actinometer and intermittent illumination apparatus (Fig. 4A).

After determining that our proposed method for the oxidation of benzhydrol catalyzed by Ru(bpy)_3_Cl_2_ is both thermodynamically and kinetically feasible (see Section N of SI), we began our studies to characterize the involvement of a possible chain reaction using our newly developed methods. Under our reaction conditions, we obtain a 77.4% conversion of benzhydrol to benzophenone after only 2 minutes of irradiation. Further details on sample preparation, and the irradiation apparatus can be found in the methods section and SI. In order to determine the Φ of this reaction, it was first necessary to determine the rate at which a 6.0 mM solution of Ru(bpy)_3_Cl_2_ absorbs photons under our geometry and conditions of irradiation. This task is greatly simplified using our newly developed Ru(bpy)_3_Cl_2_ based actinometer. By simply performing the previously described actinometry experiment with a 6.0 mM solution of Ru(bpy)_3_Cl_2_, we calculated that our sample absorbs 1.53 × 10^−7^ einsteins s^−1^. Due to the fact that a 6.0 mM solution would result in an out of scale absorption, both the initial and final solutions of the actinometer were diluted accordingly (approx. 30 fold dilution) prior to recording their absorbance at 372 nm. However, it is necessary to account for this dilution while performing the calculation as absorbance and concentration are directly proportional. Using the same molecule and concentration in the sample and actinometer eliminates issues relating to spectral and lamp matching. With this information we can simply apply equation (1) to find that the 77.4% conversion obtained in 2 minutes of irradiation corresponds to a quantum yield of 4.21 after correcting for the initiation efficiency (see Section O of SI).

To further characterize the chain reaction we have utilized our modified RS method. Using an on:off ratio of 1:2, we irradiated 10 samples, each for a total of 6 minutes (on + off time) at different flash rates. The results are displayed in Fig. 4b, in which conversion is plotted as a function of the log(*t*_on_). Qualitatively, the fact that Fig. 4b demonstrates a non-linear dependence between conversion and the rate of sample illumination reaffirms that indeed the studied reaction involves a chain mechanism. Quantitatively, we observe that there is an appreciable change in rate of conversion between 5 and 100 ms, indicating that the τ_s_ of the radical chain is somewhere between these two values. The lifetime of the chain can be approximated by using a sigmoidal curve fit, or simply determining graphically the point at which half of the total change in rate is observed. In our example (Fig. 4b) this time corresponds to 19 ms. The fact that the total conversion during fast flashing is lower than that observed during slow flashing reflects an increased number of termination events brought on by an increased concentration of propagating radicals.

## Discussion

Despite the surge of advancements in the field of visible-light mediated photoredox processes, many of these new discoveries are reported without an overall understanding of the detailed mechanism and kinetics involved. This reflects that that many synthetic laboratories lack the necessary equipment and expertise to perform these studies. One of the frequently asked questions in these studies is whether the overall mechanism involves a chain-propagating component. This is typically explored through the use of intermittent illumination, however in a significant number of reports the periods used in these experiments are in the minute timescales, which are too slow to provide any insights into a possible chain mechanism, as these occur on shorter timescales. If we put this in the same context as Fig. 4, the minutes timescale corresponds to x-scale values >5, which are in the plateau region where intermittent on-off switching would have no kinetic effect.

In order to aid synthetic laboratories in the investigation of chain reactions, we have designed a visible-light actinometer based on the ubiquitous photocatalyst, Ru(bpy)_3_Cl_2_. Coupled with well-known singlet oxygen chemistry, our actinometer system employs the sensitization of oxygen by Ru(bpy)_3_Cl_2_ to oxidize DPA to its corresponding endoperoxide. By simply monitoring the absorbance at 372 nm, the conversion of DPA over a given irradiation time can easily be determined. By carefully calibrating our actinometer with the ferrioxalate actinometer, we calculated the Φ of our system to be 0.019. The low efficiency of our system reflects the slow rate of bimolecular quenching of ^1^O_2_ by DPA. Importantly, in the actinometer Ru(bpy)_3_Cl_2_ is also a catalyst and just like in photoredox catalysis, it is not consumed during the reaction. A detailed Standard Operating Procedure is available in Section J of the Supporting Information.

In some cases, actinometry alone cannot confirm the presence of a chain mechanism, as previously discussed. To complement this technique, we have developed a revised RS method using current LED technology. Our system allows for intermittent illumination from microseconds to minutes timescales, including those relevant to typical chain propagating lifetimes, usually in the millisecond range. Moreover, our system allows precise control of the light on/light off periods, which was more difficult with the previous RS methods. We envision our technique to be used as a qualitative or semi-quantitative tool, as demonstrating a linear/non-linear dependence of the yield with illumination times and determining the lifetime of a propagating chain can be simply accomplished with this method.

Finally, we validated our new methods using the Ru(bpy)_3_Cl_2_-mediated oxidation of benzhydrol using 4-cyano-*N*-methoxypyridinium tetrafluoroborate as the chain amplifier. Using our Ru(bpy)_3_Cl_2_ actinometer, we were able to calculate a Φ of 4.21 for this reaction. Moreover, by employing our revised RS method, we were able to demonstrate a non-linear relationship between conversion and the illumination periods, and we were able to calculate Φ_s_ to be ~19 ms. With this information in hand, we can conclude that Ru(bpy)_3_Cl_2_-mediated oxidation of benzhydrol has a prominent chain-propagating component. While Φ > 4 clearly indicates the involvement of a chain reaction, this conclusion could be reached independently of the large value of Φ, as it relates to the consequence of changing the frequency of intermittent illumination only.

We envision that these methods will have great implications on the field of photoredox catalysis, as they will provide researchers with useful tools to properly characterize chain mechanisms. Our visible-light actinometer provides the advantage that the actinometer is also the photocatalyst, and our RS method is comprised of existing LED technologies while providing a smaller footprint compared to previous methods. We note that the actinometer will be useful for any process involving Ru(bpy)_3_Cl_2_, whether or not it involves photoredox catalysis.

## Methods

### General Procedure for the Ru(bpy)_3_Cl_2_ Experiments

The Ru(bpy)_3_Cl_2_ actinometer experiments were performed using a 450 nm LED in a dark room. Samples were irradiated as 3 mL samples in a precision 1 cm × 1 cm quartz cuvette and sample agitation was accomplished through the use of a mini magnetic stir bar (see Figure S9 for a photograph of the experimental apparatus). Initially, experiments performed with the aim of determining the quantum yield (Φ) for the Ru(bpy)_3_Cl_2_ mediated oxidation of 1,9-diphenylanthracene (DPA) to its corresponding endoperoxide were performed with a 440 nm notch filter (FWHM 10 nm). Once the Φ of the reaction was known the notch filter could be removed so long as there was no overlap between the absorption of Ru(bpy)_3_Cl_2_ and any of the starting materials or products of the reaction, within the LED wavelengths of irradiation. In order to determine the Φ and power dependence of the actinometer, solutions consisting of 0.194 mM Ru(bpy)_3_Cl_2_ and 0.10 mM DPA in acetonitrile were utilized. By monitoring the disappearance of the signal at 372 nm, we were able determine the amount of DPA consumed over a given period of irradiation using ε_372nm_ for DPA (11,100 M^−1^ cm^−1^). Additionally, one can determine the Φ of any Ru(bpy)_3_Cl_2_ photocatalyzed reaction by scaling the concentrations of both systems so that the concentration of Ru(bpy)_3_Cl_2_ is the same in both systems. This will ensure the same amount of photons is absorbed in both systems, allowing for easy determination of Φ. A standard operation procedure (SOP) for the Ru(bpy)_3_Cl_2_ based actinometer can be found in section J of the SI.

### General Procedure for the Ferrioxalate Actinometer Experiments

The ferrioxalate actinometer experiments were performed using a 450 nm LED equipped with a 440 nm notch (FWHM 10 nm) filter in a dark room. The samples were irradiated as 3 mL samples in a precision 1 cm × 1 cm quartz cuvette and sample agitation was accomplished through the use of a mini magnetic stir bar (see in Figure S9 a photograph of the experimental apparatus).

In performing the experiment, two solutions are required: 1) a 0.15 M potassium ferrioxalate and 2) a 0.1% buffered phenanthroline solution.

#### Preparation of 0.15 M potassium ferrioxalate

Briefly, 7.37 g of solid potassium ferrioxalate, 80 mL of H_2_O, and 10 mL of 1.0 N H_2_SO_4_ were mixed. Upon complete dissolution of the solid potoassium ferrioxalate, the solution was topped up to 100 mL with H_2_O to give a final concentration of 0.15 M.

#### Preparation of 0.1% buffered phenanthroline

Briefly, 22.5 g of sodium acetate, and 100 mg of phenanthroline were dissolved in 100 mL of 0.5 M H_2_SO_4_.

Both solutions were stored in amber bottles and used as required.

Since we are calibrating our Ru(bpy)_3_Cl_2_ based actinometer with the ferrioxalate actinometer, it is important we ensure that at the wavelengths chosen both samples absorb the same amount of light. This was achieved by placing a 440 nm notch filter (FWHM 10 nm) filter between the 460 nm LED and the samples, and ensuring the absorbance of the ferrioxalate actinometer and the Ru(bpy)3Cl2 sample was above 2 between the wavelengths of 430 and 450 nm (See Figure S3 for an overlay of the emission spectrum of the LED with the absorption spectrum of the Ru(bpy)_3_Cl_2_ and potassium ferrioxalate solutions).

In a typical experiment, two cuvettes containing 3 mL of 0.15 potassium ferrioxalate were prepared. One sample was irradiated for 1 minute using the LED, while the other was left in the dark as a control. Upon completion of irradiation, 500 μL of the 0.1% buffered phenanthroline solution was added to both of the samples. The samples were then allowed to develop in the dark for another 5 minutes before the absorption of each of the samples were measured at 510 nm. Using the optical difference (ΔA_510 nm_) between the irradiated and control (dark) sample and the ε_510 nm_ = 11,100 M^−1^cm^−1^, the amount of Fe^2+^ produced during the irradiation can be determined. Knowing that the quantum yield for Fe^2+^ production is 1.01 and that the samples absorbs >99% of the incident light, one can easily calculate the photon flux absorbed by the sample (See Section F of SI for more information regarding the calculations).

### General Procedure for Ru(bpy)_3_Cl_2_ Laser Flash Photolysis Experiments

The triplet quenching experiments of Ru(bpy)_3_Cl_2_ were performed using a Nd-YAG laser (355 nm and 10 mJ/pulse) in a LFP-111 laser flash photolysis sytem (Luzchem Inc., Ottawa, Canada) and 1 × 1 cm quartz cuvettes. Samples of Ru(bpy)_3_Cl_2_ were prepared in acetonitrile with a total volume of 3 mL and an absorbance of 0.1 at 355 nm. The samples were deaerated with N_2_ 30 minutes prior to use. A stock solution of 5 mM DPA used for the quenching studies was also prepared in acetonitrile. Experiments probing the sensitization of DPA by ^3^Ru(bpy)_3_Cl_2_ were performed using a Surelite plus OPO (460 nm and 10 mJ/pulse) as to avoid direct excitation of the DPA.

### General Procedure for Singlet Oxygen Laser Flash Photolysis Experiments

The singlet oxygen (^1^O_2_) quenching experiments by DPA were performed using a Nd-YAG laser (532 nm and 10 mJ/pulse) in a LFP-111 laser flash photolysis system (Luzchem Inc., Ottawa, Canada) and 1 × 1 cm quartz cuvettes fitted with a Hamamatsu NIR-PMT which monitored the phosphorescence of ^1^O_2_ at 1270 nm. Excitation of Rose Bengal in 1 mL of CD_3_CN at 532 nm was used to sensitize the production of ^1^O_2_. A solution of 5 mM DPA prepared in CD_3_CN was used as stock solution for the quenching experiments.

### General Procedure for the Oxidation of Benzhydrol

Typically, 4-cyano-*N-*methoxypyridinium tetrafluoroborate (0.09 mmol, 20.0 mg), benzhydrol (0.09 mmol, 16.6 mg), Ru(bpy)_3_Cl_2_ (0.018 mmol, 13.5 mg), and acetonitrile (3 mL) were added to a 1 cm × 1 cm quartz cuvette fitted with a septa. The reaction mixture was then degassed with argon for 15 minutes. It was then subjected to 460 nm LED irradiation for 2 minutes. Upon completion of the irradiation, the reaction mixture was concentrated using a rotovap. The percent conversion was then determined using ^1^H-NMR, by monitoring the disappearance of the methoxy signal belonging to the pyridium salt and using dimethylsulfone as external standard.

### General Procedure for “Rotating Sector” Experiments for the Oxidation of Benzhydrol

Typically, 4-cyano-*N-*methoxypyridinium tetrafluoroborate (0.09 mmol, 20.0 mg), benzhydrol (0.09 mmol, 16.6 mg), Ru(bpy)_3_Cl_2_ (0.018 mmol, 13.5 mg), and acetonitrile (3 mL) were added to a 1 cm × 1 cm quartz cuvette fitted with a septa. The reaction mixture was then degassed with argon for 15 minutes before it was intermittently irradiated for 6 minutes (light on + off time) using a pulsed 460 nm LED, which was powered by a constant current driver (designed and built in house) and controlled by a digital delay/pulse generator (Stanford Research System Inc.- MODEL DG535). In all cases the system was interfaced with an oscilloscope (Tektronix–MODEL TDS3052), which monitored the delivered voltage and resulting current of the system. The system was also interfaced with a photodiode, which allowed us to monitor the shape and duration of the light pulse emitted from the LED. This allowed us to monitor the light pulse in real time to ensure that the appropriate light on:light off ratio was being employed. A light on:light off ratio of 1:2 was used in all trials, and the length of the on and off times were increased proportionaly with each successive trial. After the irradiation, the reaction mixture was concentrated using a rotovap. The percent conversion was then determined using ^1^H-NMR, by monitoring the disappearance of the methoxy signal belonging to the pyridium salt and using dimethylsulfone as external standard.

## Additional Information

**How to cite this article**: Pitre, S. P. *et al.* Visible-Light Actinometry and Intermittent Illumination as Convenient Tools to Study Ru(bpy)_3_Cl_2_ Mediated Photoredox Transformations. *Sci. Rep.* **5**, 16397; doi: 10.1038/srep16397 (2015).

## Supplementary Material

Supplementary Information

## Figures and Tables

**Figure 1:**
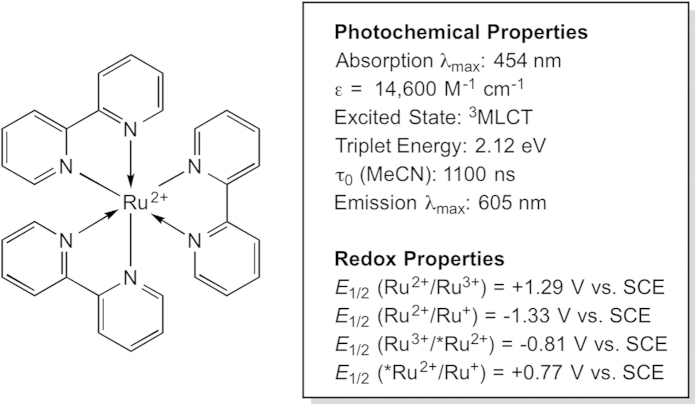
The photochemical and redox properties of the popular Ru(bpy)_3_Cl_2_ photocatalyst^1^.
