# Supplementary material for: Visible-Light Actinometry and Intermittent Illumination as Convenient Tools to Study Ru(bpy)3Cl2 Mediated Photoredox Transformations
Source: Sci Rep. 2015 Nov 18;5:16397. doi: 10.1038/srep16397 (PMC4649705; doi:10.1038/srep16397)
Supplement: Supplementary Information [file srep16397-s1.pdf]

Supplementary Data

Visible-Light Actinometry and Intermittent Illumination as Convenient Tools to Study $Ru(bpy)_3Cl_2$ Mediated Photoredox Transformations

By Spencer P. Pitre, Christopher D. McTiernan, Wyatt Vine, Rebecca DiPucchio, Michel Grenier, and Juan C. Scaiano

Email: scaiano@photo.chem.uottawa.ca

**A.**	**General Information**	**S2**
**B.**	**UV-Vis spectrum of 0.1 mM DPA in CH_3CN**	**S2**
**C.**	**Absorbance @ 372 nm as a function of [DPA] for determination of ε of DPA**	**S3**
**D.**	**Preparation of potassium ferrioxalate**	**S4**
**E.**	**Overlay of the emission spectrum of the 460 nm LED and 460 nm LED fitted with a 440 nm notch filter (FWHM 10 nm) with the absorption spectrum of the $Ru(bpy)_3Cl_2$ and potassium ferrioxalate solutions**	**S4**
**F.**	**Calculating the rate of photon absorption by the ferrioxalate actinometer using the 460 nm LED fitted with a 440 nm notch filter (FWHM 10 nm)**	**S4**
**G.**	**Power dependence of $Ru(bpy)_3Cl_2$ based actinometer**	**S6**
**H.**	**Quenching of $^1O_2$ by 1,9-diphenylanthracene**	**S7**
**I.**	**Sensitization of 1,9-diphenylanthracene by $^3Ru(bpy)_3Cl_2$**	**S8**
**J.**	**End user instructions (SOP)**	**S9**
**K.**	**History and theory behind the rotating sector method**	**S11**
**L.**	**Photographs of the irradiation apparatus used for both steady-state and intermittent illuminations**	**S14**
**M.**	**Synthesis of 4-cyano-*N*-methoxypyridinium tetrafluoroborate**	**S12**
**N.**	**Thermodynamics and kinetics of the $Ru(bpy)_3Cl_2$ mediated oxidation of benzhydrol**	**S13**
**O.**	**Further examination of the quantum yield for the $Ru(bpy)_3Cl_2$ mediated oxidation of benzhydrol in acetonitrile**	**S14**
**P.**	**Proposed mechanism for the $Ru(bpy)_3Cl_2$ mediated oxidation of benzhydrol**	**S16**
**Q.**	**^1H and ^{13}C NMR Spectra**	**S20**
**R.**	**References**	**S34**

A. General Information

Tris(bipyridine)ruthenium(II) chloride ($Ru(bpy)_3Cl_2$) was purchased from Fisher Scientific and used as received. Diphenylanthracene (DPA), benzhydrol, 4-cyanopyridine *N*-oxide, and trimethyloxonium tetrafluoroborate were purchased from Sigma Aldrich and used as received. Flash column chromatography was performed using 230-400 mesh silica gel. All ^1H and ^{13}C NMR were recorded on a Bruker AVANCE 400 spectrometer. Chemical shifts (δ) are reported in ppm from the solvent resonance as the internal standard ($CDCl_3$: δ 7.26 ppm).

B. UV-Vis spectrum of 0.1 mM DPA in CH_3CN

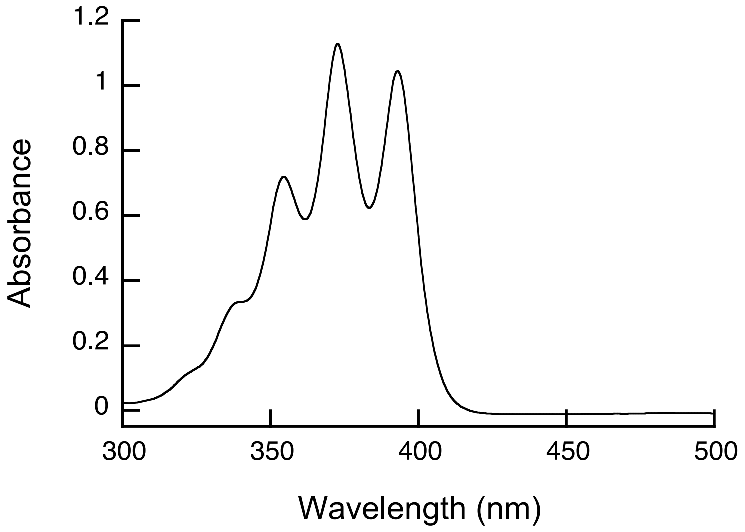

Figure S1: UV-Vis Spectrum of 0.10 mM DPA in acetonitrile

C. Absorbance @ 372 nm as a function of [DPA] for determination of ε of DPA

Through determination of the ε for DPA in acetonitrile at 372 nm, we can correlate the change in absorption with the number of moles of DPA actually consumed.

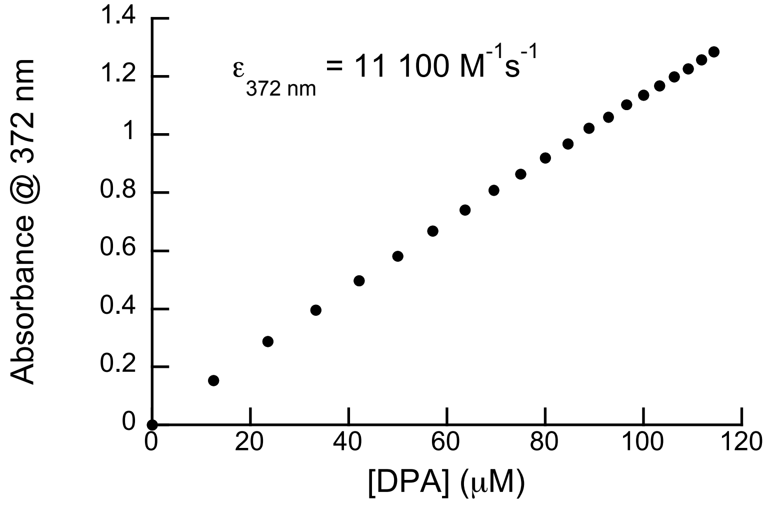

Figure S2: Absorbance @ 372 nm as a function of [DPA] for determination of ε of DPA

D. Preparation of potassium ferrioxalate

The solid potassium ferrioxalate was prepared as described by Hatchard, C.G.; Parker, C.A. *Proc. Roy. Soc. (London)* **1956**, *A235*, 518-536. Briefly, 1.5 M potassium oxalate and 1.5 M ferric chloride were prepared in Milli-Q H_2O. The two solutions were then combined under stirring in a 3:1 ratio (potassium oxalate:ferric chloride). After letting the solution stir for 2 hours the resulting precipitate was filtered off and recrystallized three times from warm water. The solid was then allowed to dry under vacuum and was stored in the dark.

E. Overlay of the emission spectrum of the 460 nm LED and 460 nm LED fitted with a 440 nm notch filter (FWHM 10 nm) with the absorption spectrum of the $Ru(bpy)_3Cl_2$ and potassium ferrioxalate solutions

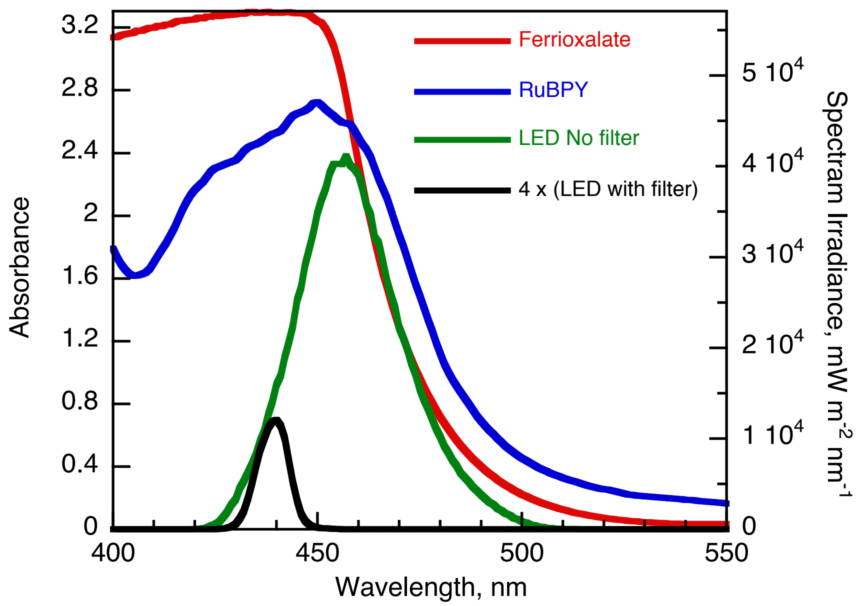

Figure S3: Overlay of the emission spectrum of the 460 nm LED and 460 nm LED fitted with 440 nm notch filter (FWHM 10 nm) with the absorption spectrum of the $Ru(bpy)_3Cl_2$ and potassium ferrioxalate solutions.

F. Calculating the rate of photon absorption by the ferrioxalate actinometer using the 460 nm LED fitted with a 440 nm notch filter (FWHM 10 nm)

In order to determine the effective intensity of the 460 nm LED fitted with a 440 nm notch filter (FWHM 10 nm) we utilized the ferrioxalate actinometer, which has been described in detail in the online methods section.

From the previously described experiment we have found the following results after 1 minute of irradiation:

Table S1: Results from ferrioxalate actinometry experiment. Samples irradiated for 1 minute as 3 mL samples in 1 cm x 1 cm quartz cuvettes. [potassium ferrioxolate] = 0.15 M. Solutions developed with 500 μL of 0.15 buffered phenanthroline.

	**Absorption of sample @ 510 nm**		
	Irradiated sample (A_{light})	Dark sample (A_{dark})	A_{light} - A_{dark}
	1.402	0.121	1.281
	1.400	0.134	1.266
	1.381	0.148	1.233
Average	1.394	0.134	1.26

From this we can determine the concentration of ferrous ions (Fe^{2+}) produced during the the 1 minute irradiation as follows:

$$[Fe^{2+}] = \frac{A_{light} - A_{dark}}{\varepsilon_{510\ nm}\ l}$$

$$[Fe^{2+}] = \frac{1.394 - 0.134}{11\ 100\ M^{-1}cm^{-1}\ (1\ cm)}$$

$$[Fe^{2+}] = 1.135 \times 10^{-4}\ M$$

Since we know the volume of the sample to be 3.5 mL we can find the total number of moles Fe^{2+}.

$$\#\ of\ moles\ Fe^{2+} = 3.5 \times 10^{-3}\ L\ (1.135 \times 10^{-4}\ M)$$

$$\#\ of\ moles\ Fe^{2+} = 3.97 \times 10^{-7}\ mol$$

Lastly we can apply the following equation to determine the number of photons absorbed by our sample per unit time.

$$\frac{Nh\nu}{t} = \frac{\text{moles of } Fe^{2+}}{\Phi\ t\ F}$$

$$\frac{Nh\nu}{t} = \frac{3.405 \times 10^{-7}\ mol}{(1.01)(60\ s)(1)}$$

$$\frac{Nh\nu}{t} = 6.5 \times 10^{-9}\ mol\ h\nu\ s^{-1}$$

From this we know that any sample with an absorbance above 2 within the wavelengths of irradiation will absorb 6.5×10^{-9} mol hν s^{-1} if it is irradiated using the same geometry.

G. Power dependence of $Ru(bpy)_3Cl_2$ based actinometer

To be useful with a variety of different illumination sources the actinometer should respond linearly to increasing powers of visible light illumination. For this reason we have examined the $Ru(bpy)_3Cl_2$ based actinometer using the 460 nm LED at different irradiances. Through modulation of the systems current we have been able to adjust the power of the LED. Powers were measured using a Luzchem SPR-4001 spectroradiometer.

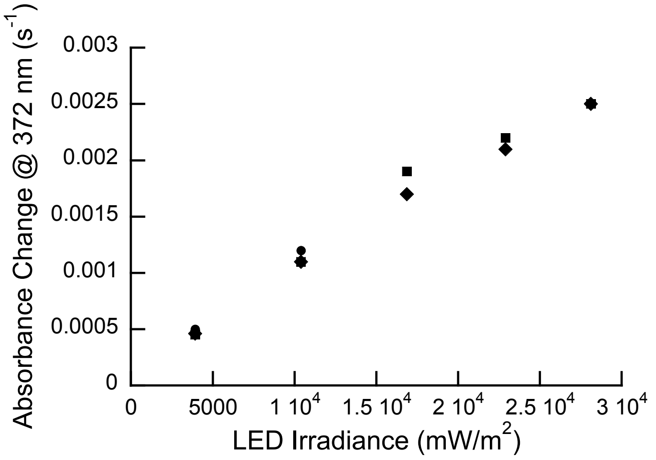

Figure S4: Rate of the change in absorption @ 372 nm as a function of 460 nm LED irradiance. Trial 1 (•), Trial 2 (■), and Trial 3 (◆)

H. Quenching of $^1O_2$ by 1,9-diphenylanthracene

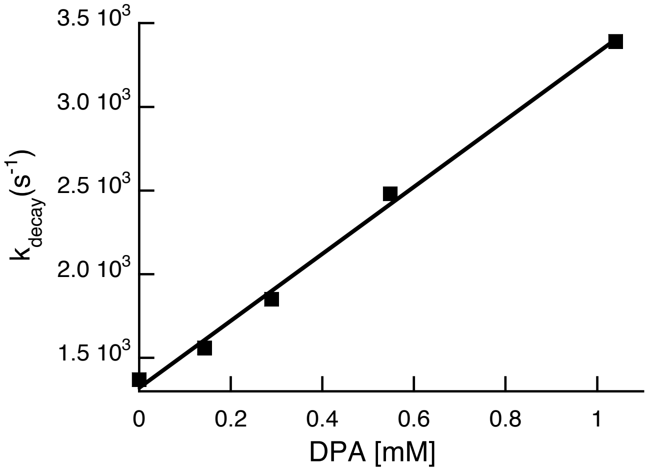

Figure S5: Quenching of $^1O_2$ by 1,9-diphenylanthrancene (DPA) in CD_3CN. The bimolecular rate constant corresponds to the slope of this plot.

I. Sensitization of 1,9-diphenylanthracene by $^3Ru(bpy)_3Cl_2$

Through the use of LFP one can demonstrate the sensitization of DPA by triplet $^3Ru(bpy)_3Cl_2$ even though the triplet-triplet absorption of DPA (λ_{max} 440 nm) and ground-state absorption of the $Ru(bpy)_3Cl_2$ overlap. This is due to the fact that ^{3}DPA has a relatively large ε and a τ that is considerably longer than that of $^3Ru(bpy)_3Cl_2$, making it possible to observe the growth and decay of the signal corresponding to ^{3}DPA upon 460 nm laser (10 mJ per pulse) excitation of a dearated solution of $Ru(bpy)_3Cl_2$ and DPA in CH_3CN. As can be seen in Figure S6, in the absence of DPA (black trace) one observes only bleaching of $Ru(bpy)_3Cl_2$ at 440 nm. However, upon addition of DPA (blue trace) there is a clear growth and decay of a signal that we attribute to ^{3}DPA.

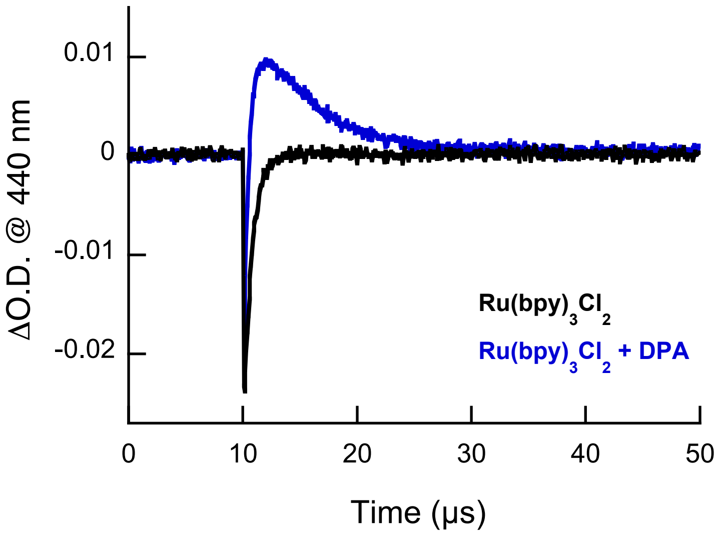

Figure S6: LFP traces obtained upon 460 nm (10mJ per pulse) excitation of a dearated solution of $Ru(bpy)_3Cl_2$ (**black**) and $Ru(bpy)_3Cl_2$ + 1,9-diphenylanthrancene (**blue**) in CH_3CN while monitoring at 440 nm.

J. End user instructions (SOP)

1) Due to the visible light nature of the $Ru(bpy)_3Cl_2$ actinometer, it is important that experiments be performed in a dark room as to avoid conversion due to ambient lighting.
2) Ensure that the geometry of irradiation of both the actinometer and reaction of interest is consistent.
   I. Utilize a sample holder which allows for reproducible sample placement
   II. Secure both the sample holder and light source to ensure fixed distance
   III. Utilize the same reaction vessel or perfectly matched pairs for all measurements
3) As the analysis of the actinometer is done spectrophotometrically, it may be advantageous to perform the irradiation in a cuvette. However, this is not necessary, as in many cases the samples will require dilution prior to analysis.
4) Prepare a solution of $Ru(bpy)_3Cl_2$ (same concentration as that used in the reaction of interest) and DPA (0.10 mM) in acetonitrile. As $Ru(bpy)_3Cl_2$ is both the photocatalyst and actinometer, this is a simple way to match lamp-sample spectral overlap.

   Important: The accuracy of the actinometer relies on this concentration match.
5) Record a UV-Vis spectra of the $Ru(bpy)_3Cl_2$ and DPA solution. This will be your "time zero" spectrum.
6) As the absorption of $Ru(bpy)_3Cl_2$ and DPA at 372 nm is additive, depending on the concentration of $Ru(bpy)_3Cl_2$ it may be necessary to dilute the sample for analysis to ensure that the absorption is within the linear response region of the spectrophotometer (see the specifications of your spectrophotometer). Most spectrometers do not perform well at absorbances above 2.5.
7) Note the absorbance at 372 nm ($A_{INITIAL}$).
8) Irradiate the sample for a set period of time.
9) Record a new UV-Vis Spectrum of the irradiated sample and note the absorbance at 372 nm (A_{FINAL}).
10) As $Ru(bpy)_3Cl_2$ should not photobleach on the timescale of the irradiation, there will be a residual absorption at 372 nm, even when all DPA has been consumed. For this reason, A_{FINAL} will be larger than the absorbance found at 372 nm in the absence of DPA.
11) Knowing the volume of the irradiated sample and that the molar extinction coefficient (ε) for DPA is 11,100 $M^{-1}cm^{-1}$ one can determine the number of moles of DPA consumed during the irradiation using equation (S.1).

$$\#\ \text{of moles DPA consumed} = \left(\frac{A_{INITIAL} - A_{FINAL}}{\varepsilon_{372\ nm}\, l}\right) \times V \qquad (S.1)$$

Where $A_{INITIAL}$ and A_{FINAL} are the absorbance of the solution at 372 nm before and after irradiation, respectively; $\varepsilon_{372\ nm}$ is the extinction coefficient of DPA at 372 nm in acetonitrile, l is the path length of the cuvette, and V is the volume of the sample for which the absorption was measured. If a dilution was made before the measurement this must be taken into account by applying standard volumetric dilution factors.

- Since the quantum yield (Φ) for the actinometer is known to be 0.019,[ξ] we can then use the number of moles DPA consumed to determine the number of moles of photons absorbed by our sample per unit time ($\frac{Nh\nu}{t}$) by applying equation (S.2).

$$\frac{Nh\nu}{t} = \frac{\text{moles of DPA consumed}}{\Phi\ t} \qquad (\text{S}.2)$$

  where Φ is the quantum yield of DPA consumption, and *t* is the irradiation time in seconds.

- To determine the Φ for the reaction of interest you must then irradiate the sample of interest using the same geometry employed for the actinometer. Once irradiation is complete you must then determine the number of moles of product formed or substrate consumed per unit time using your favourite analytical technique. From this one can apply equation (S.3) to determine the Φ of their reaction.

$$\Phi = \frac{\text{\# of moles consumed or produced}}{t} \times \left(\frac{Nh\nu}{t}\right)^{-1} \qquad (\text{S}.3)$$

  Where t is the time of irradiation and $\left(\frac{Nh\nu}{t}\right)^{-1}$ is the reciprocal of the number of photons absorbed by the sample per unit time.

[ξ] This quantum yield was determined in Ottawa, Canada where the elevation is 70 m. Dramatic changes in elevation could have a modest effect in the amount of oxygen dissolved and cause a slight reduction of this value.

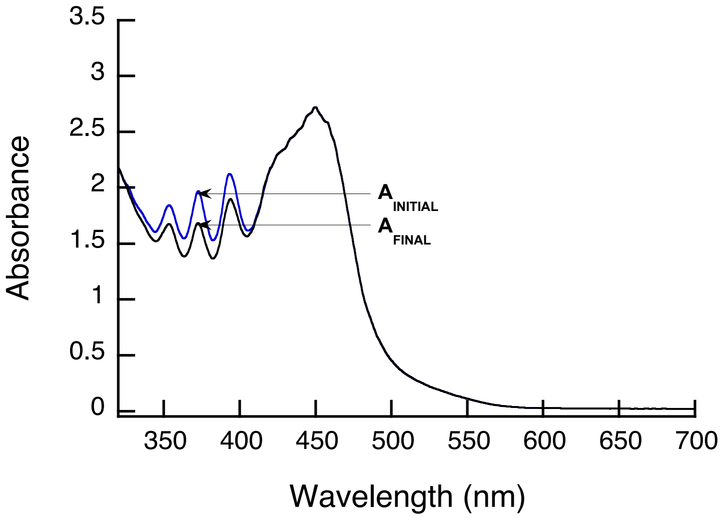

Figure S7: Absorption spectra of a typical actinometry experiment performed with $Ru(bpy)_3Cl_2$ (0.19 mM) and DPA (0.10 mM) in acetonitrile and irradiated with a 460 nm LED. $A_{INITIAL}$ is before irradiation and A_{FINAL} is after 6 minutes of irradiation.

In some cases it may be necessary to perform the actinometer and the reaction of interest on different time scales and then correct for the time of exposure.

K. History and theory behind the rotating sector method

The use of intermittent illumination in photo-initiated reactions is in no way a new idea, having been initially suggested in 1929 by Briers, Chapman and Walters.[1] However, it was not until 1946 when Burnett and Melville expanded on the theory of intermittent illumination and applied it to the direct photo-polymerization of vinyl acetate that it became evident one could use such intermittent illumination to characterize chain reactions, in particular the average lifetime of a chain sequence.[2] The technique has since become known as the "rotating sector" method as at the time the simplest route to intermittent illumination was to place a rotating sectored disk in the path of the light source. Using this set-up, one could control the light/dark ratio by controlling the size and/or number of sectors in the disk as well as the duration of the light-on and light-off periods through its speed of rotation.[2] Over the next few decades the theory and the instrumentation behind the technique expanded, as it was a relatively simple technique for obtaining kinetic data for photochemically initiated chain reactions exhibiting bimolecular chain termination. The technique eventually fell out of favour as it was demonstrated that electron spin resonance (ESR) spectroscopy could measure the rates of bimolecular radical reactions involved in both terminating and non-terminating chain events, some of which were only previously accessible through the use of intermittent illumination.[3]

Although the idea of utilizing a rotating sectored disk to intermittently irradiate a sample seems simple in principle, in practice its implementation is met with many difficulties the

least of which is the procurement of a functioning rotating sector apparatus. Other challenges in its operation include its large physical footprint, the requirement of two different sectored disks (one for short pulses and one for long pulses),[4] and poor control of the speed of rotation. High frequency of exposure also becomes difficult as fast rotating disks present safety risks.

In a typical rotating sector experiment the light is interrupted in such a way that the period of irradiation (t_{on}) is followed by an off time (t_{off}) that is equal to or longer than that of t_{on}. When the flashing rate is slow, t_{on} is much longer than the radical lifetime and because of this the radical concentration quickly increases to the value reached during steady-state irradiation $[\mathrm{R}\bullet]_{\mathrm{SS}}$. However, since the period over which the radicals decay is short in comparison to the dark period (t_{off}), during the slow flashing cycle the radical concentration drops off to essentially zero whenever the light is off. The average concentration of radicals $[\mathrm{R}\bullet]_{\mathrm{Avg}}$ in the system throughout the experiment during slow flashing would thus be $1/2[\mathrm{R}\bullet]_{\mathrm{SS}}$ if the period of light on and off were equal. Such a situation is depicted in Figure S8a. On the other hand if the flashing rate is increased to the point where t_{on} is significantly shorter than τ_{s} the situation is much different. Under these conditions the radicals initiated during a single t_{on} period will continue to grow through several successive on-off cycles until they eventually level off. Since the radical concentrations does not reach the extreme high and low values it does when the flashing rate is slow, the $[\mathrm{R}\bullet]_{\mathrm{Avg}}$ of the system approaches the steady-state concentration $[\mathrm{R}\bullet]_{\mathrm{SS}}$ when the illumination is uninterrupted (Figure S8b). From the plots in Figure S11, it becomes evident that as long as the intensity of irradiation and total irradiation time are kept constant, one should see a difference in the average rate of reaction for the fast and slow flashing experiments with the change in rate becoming apparent as the t_{off} time reaches that of the radical chain lifetime (τ_{s}).

One of the simplest ways to observe the change in reaction rate when moving from fast to slow flashing is to plot the percent conversion of the reaction versus log(t_{on}) of the flashing cycle. The first point of inflection in this plot corresponds to the average chain lifetime (τ_{s}). Although it may also be interesting to compare the conversions obtained under intermittent illumination with those obtained using steady-state illumination, it is not a requirement in the determination of τ_{s}. In doing so it is also important to account for the fact that intermittently illuminated samples are only irradiated for one-half or one-third of the experiment depending on the on/off ratio used and therefore this should also be accounted for in the constant illumination of samples. It may also be important to monitor sample temperature as constant illumination may result in temperature increases, which may influence the observed rate of reaction.

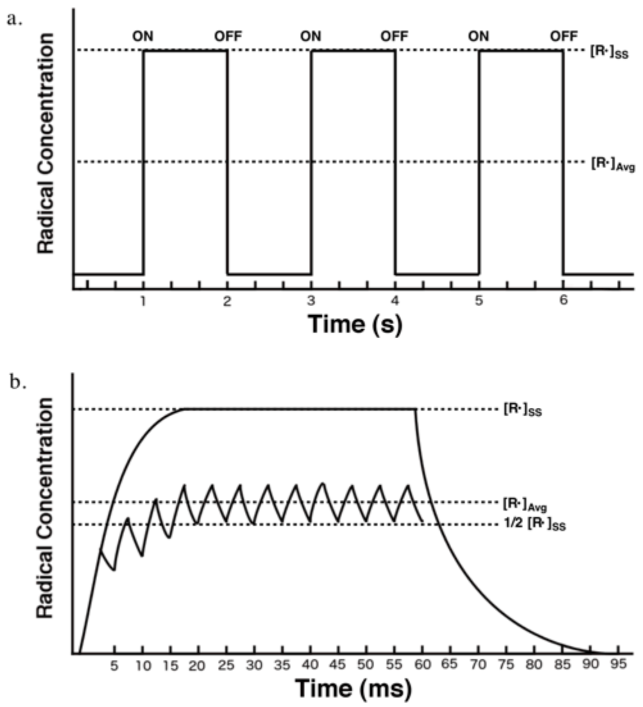

Figure S8: Radical concentration versus time during intermittent illumination (a) Slow flashing, $t_{on} >> \tau_s$. (b) Fast flashing, $t_{on} \leq \tau_s$.[3]

L. Photographs of the irradiation apparatus used for both steady-state and intermittent illuminations

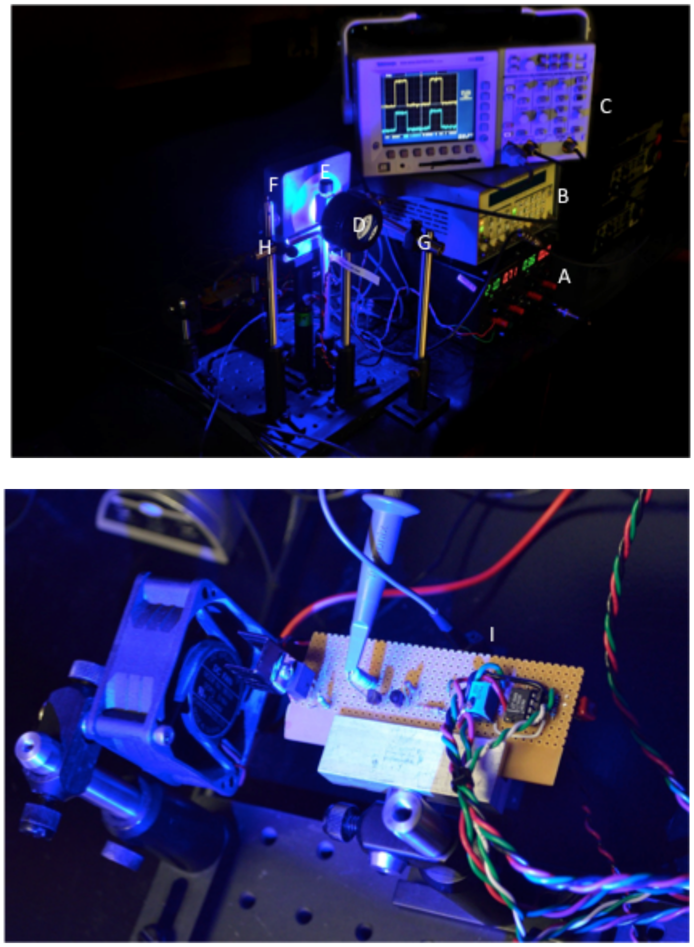

Figure S9: Photographs of the irradiation apparatus used for both steady-state and intermittent illuminations. A) power supply, B) pulse generator, C) Oscilloscope, D) 460 nm LED, E) sample holder, F) magnetic stir plate, G) photodiode, H) removable notch filter, and I) constant current driver.

M. Synthesis of 4-cyano-*N*-methoxypyridinium tetrafluoroborate

An oven-dried 100 mL round bottom flask was charged with 4-cyanopyridine *N*-oxide (6 mmol, 720 mg) and trimethyloxonium tetrafluoroborate (11.37 mmol, 1.68 g). The contents were dissolved in 30 mL of dry DCM, and the reaction mixture was purged with argon for 15 minutes and stirred overnight. The reaction was quenched with 15 mL of MeOH, and the solvent was removed under reduced pressure. The crude solid was recrystallized in MeOH to afford 4-cyano-N-methoxypyridium tetrafluoroborate as a white crystalline solid (910 mg, 68% isolated yield).

^{1}H NMR (400 MHz, DMSO-d_6) δ ppm 4.45 (s, 3 H) 8.64 - 8.98 (m, 2 H) 9.58 - 9.89 (m, 2 H)

^{13}C NMR (101 MHz, DMSO-d_6) δ ppm 70.04, 114.94, 126.39, 132.93, 142.59

Reference: Shukla, D.; Ahearn, W. G.; Farid, S. *J. Org. Chem.* **2005**, *70*, 6809-6819.

N. Thermodynamics and kinetics of the Ru(bpy)$_3$Cl$_2$ catalyzed oxidation of benzhydrol

If we calculate the Gibbs free energy for electron transfer between *Ru(bpy)$_3$Cl$_2$ and 4-cyano-*N*-methoxypyridinium tetrafluoroborate, we find that the initial electron transfer between *Ru(bpy)$_3$Cl$_2$ [E^{ox} (Ru^{3+}/*Ru^{2+}) = -0.81 V vs. SCE] and the pyridinium salt [E^{red} salt -0.50 V vs. SCE] is exothermic by 0.31 eV. While this indicates that the initial excited state electron transfer is thermodynamically possible, it does not confirm that it is also kinetically favorable. Through the use of LFP, we have also determined that 4-cyano-*N*-methoxypyridinium tetrafluoroborate quenches *Ru(bpy)$_3$Cl$_2$ at a rate of 6.9 x 10^8 M^{-1}s^{-1} (See Figure S10). Although we would expect the electron transfer to be diffusion controlled since it is exothermic by more than 0.2 eV, the potential of the pyridium salts is only an estimate based on its corresponding *N*-alkyl analogue. We have also determined that the lifetime of Ru(bpy)$_3$Cl$_2$ remains unchanged in the presence of benzhydrol. With this information in hand, the percentage of ^{3}Ru(bpy)$_3$Cl$_2$ quenched by the pyridinium salt under initial reaction conditions can be calculated using the following equation:

$$\%\,^{3}\mathrm{Ru(bpy)}_3^{2+}\ \text{quenched by Salt} = \frac{100\% \times \mathrm{k_q^{Salt}[Salt]}}{(\tau_0^{-1} + \mathrm{k_q^{Salt}[Salt]}\,)}$$

From this we calculate that 89.7.% of Ru(bpy)$_3$Cl$_2$ triplets are quenched by the pyridinium salt under initial reaction conditions. When we combine this with the fact that the efficiency of intersystem crossing in Ru(bpy)$_3$Cl$_2$ nearly unity ($\eta_{ISC} \approx 1$), we find that the initiation efficiency of the reaction is approxamitely 0.90.

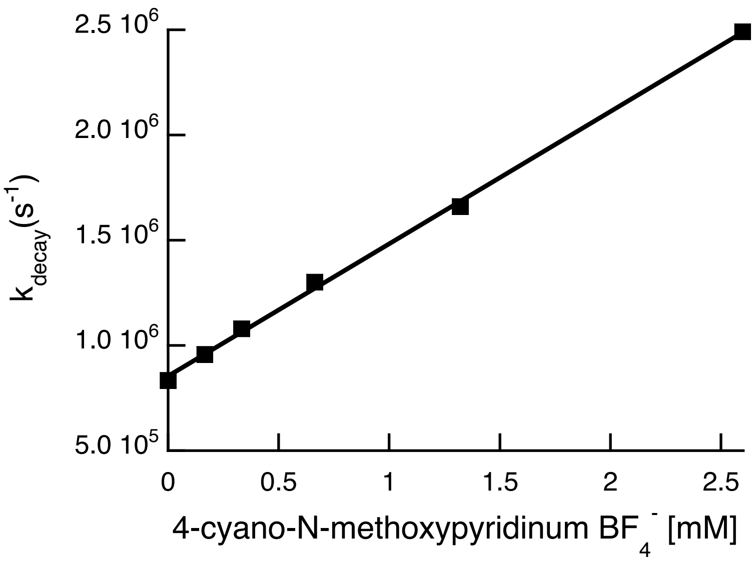

Figure S10: Quenching of *Ru(bpy)$_3$Cl$_2$ by 4-cyano-*N*-methoxypyridinum tetrafluoroborate in acetonitrile. The bimolecular rate constant corresponds to the slope of this plot.

O. Further examination of the quantum yield for the Ru(bpy)$_3$Cl$_2$ mediated oxidation of benzhydrol

We have explored the dependency of the quantum yield on the power of irradiance, as well as the concentration of benzhydrol and 4-cyano-*N*-methoxypyridinium tetrafluoroborate. As the intensity of the absorbed light increases we have found that the quantum yield of the reaction decreases, and that there is a relationship between the square root of the light intensity and the quantum yield of the chain (See Figure S11). Although such a result would be unexpected in a direct photolysis experiment under photosensitized conditions this is anticipated. It is also seen that the quantum yield is independent of benzhydrol concentration but dependent on the concentration of the pyridinium tetrafluoroborate. Unexpectedly in the presence of 60 mM 4-cyano-*N*-methoxypyridinium tetrafluoroborate the conversion over 2 minutes is reduced to 43.2%. One would expect an increase in the concentration of the pyridinium salt to result in a decrease in termination events and therefore an increase the yield. A possible explanation for this observation is that the BF_4^- anion of the pyridinium salt is dynamically quenching or displacing the Cl^- counter-ion of the Ru(bpy)$_3^{2+}$ resulting in a drastically reduced excited state lifetime, which would ultimately decrease the initiation efficiency (see Figure S12).

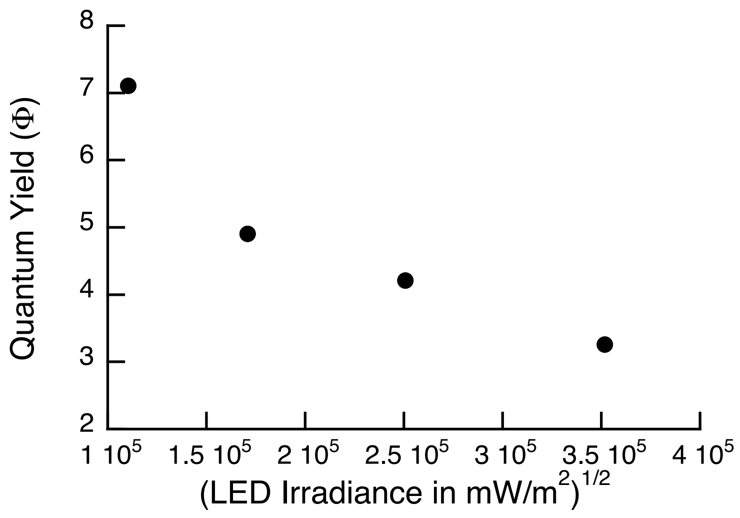

Figure S11: Quantum Yield of the Ru(bpy)$_3$Cl$_2$ mediated oxidation of benzhydrol to benzophenone as a function of irradiation power (irradiance).

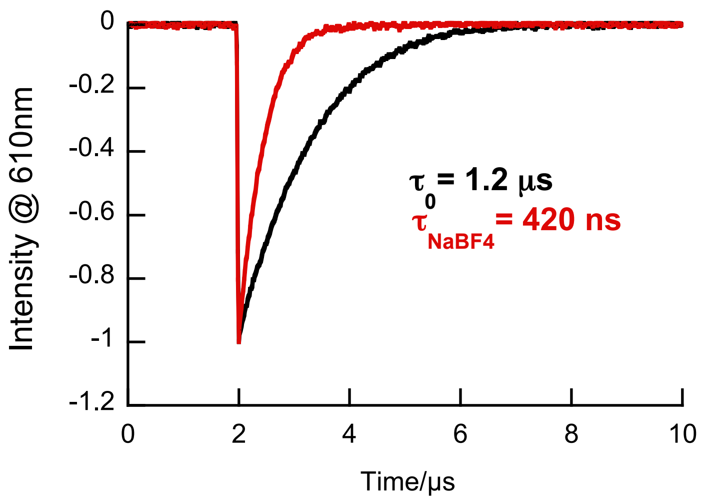

Figure S12: Decay trace of ^{3}Ru(bpy)$_3$Cl$_2$ @ 610 nm in N_2 degassed acetonitrile, in the presence of 0 mM $NaBF_4$ (black) and 0.03 M $NaBF_4$ (red)

P. Proposed mechanism for the $Ru(bpy)_3Cl_2$ mediated oxidation of benzhydrol in acetonitrile

Initiation

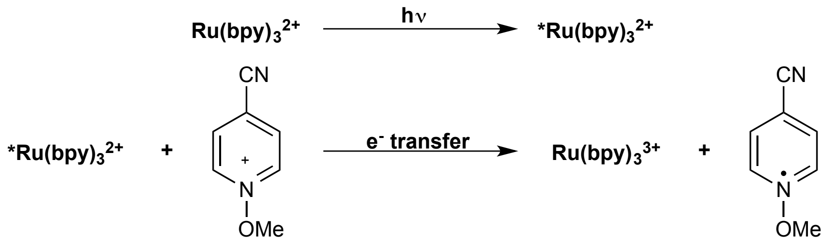

Propagation

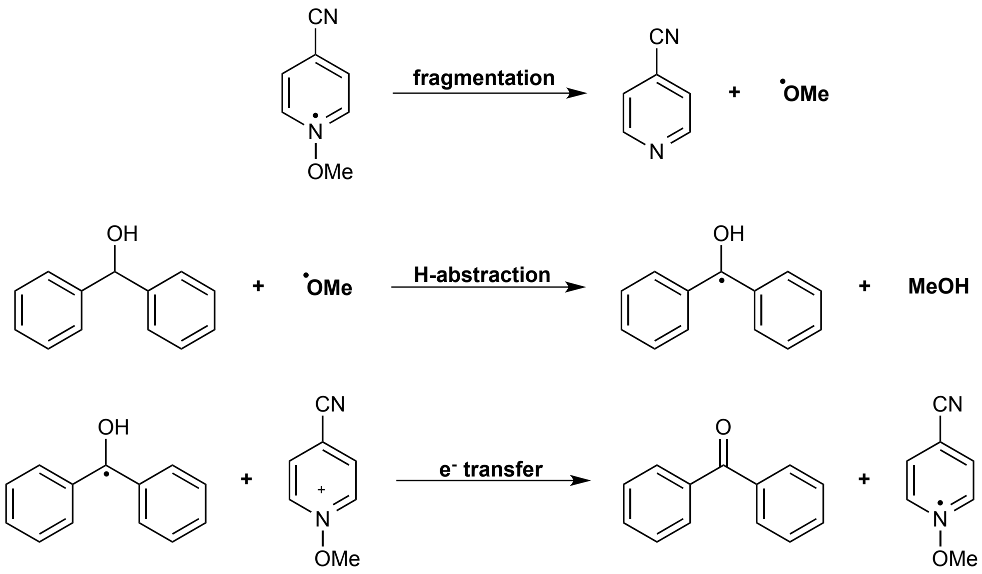

Plausible Termination

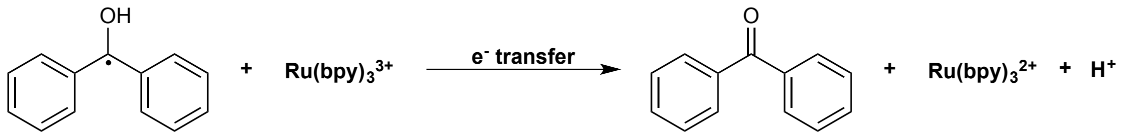

Note: The oxidation potential (E^{ox}) of benzhydrol is > 2.04 V vs. SCE.[5] Since the reduction potential of excited state $^*Ru(bpy)_3^{2+}$ is [E^{red} (*II/I) = + 0.77 V vs. SCE] and that of oxidized $Ru(bpy)_3^{3+}$ is [E^{red} (III/II) = + 1.29 V vs. SCE], electron transfer from benzhydrol to either $^*Ru(bpy)_3^{2+}$ or $Ru(bpy)_3^{3+}$ is thermodynamically unfavourable.

Q. ^{1}H and ^{13}C NMR Spectra

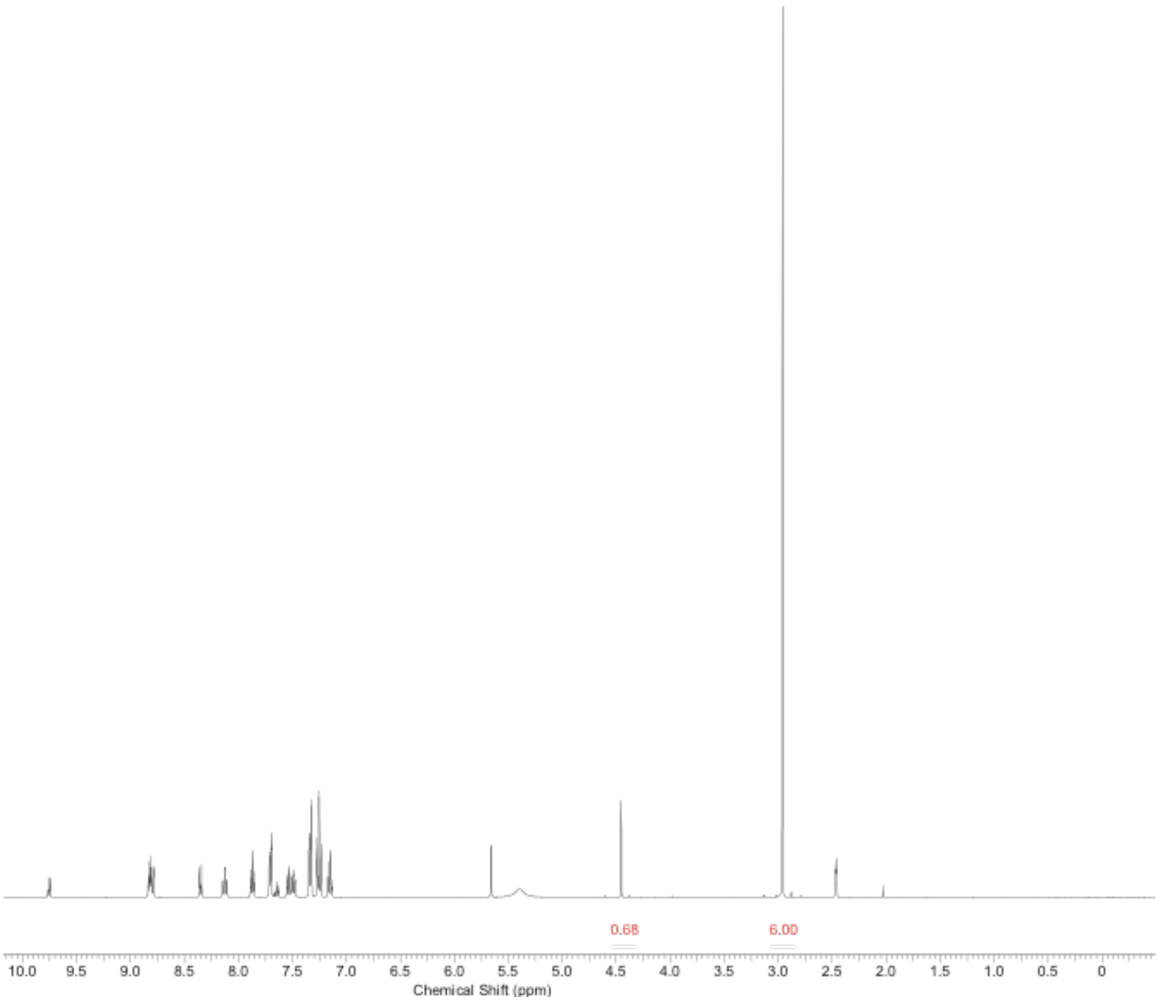

Figure S13: ^{1}H-NMR obtained after 2 min steady state irradiation [Ru(bpy)$_3$Cl$_2$ mediated oxidation of benzhydrol to benzophenone]

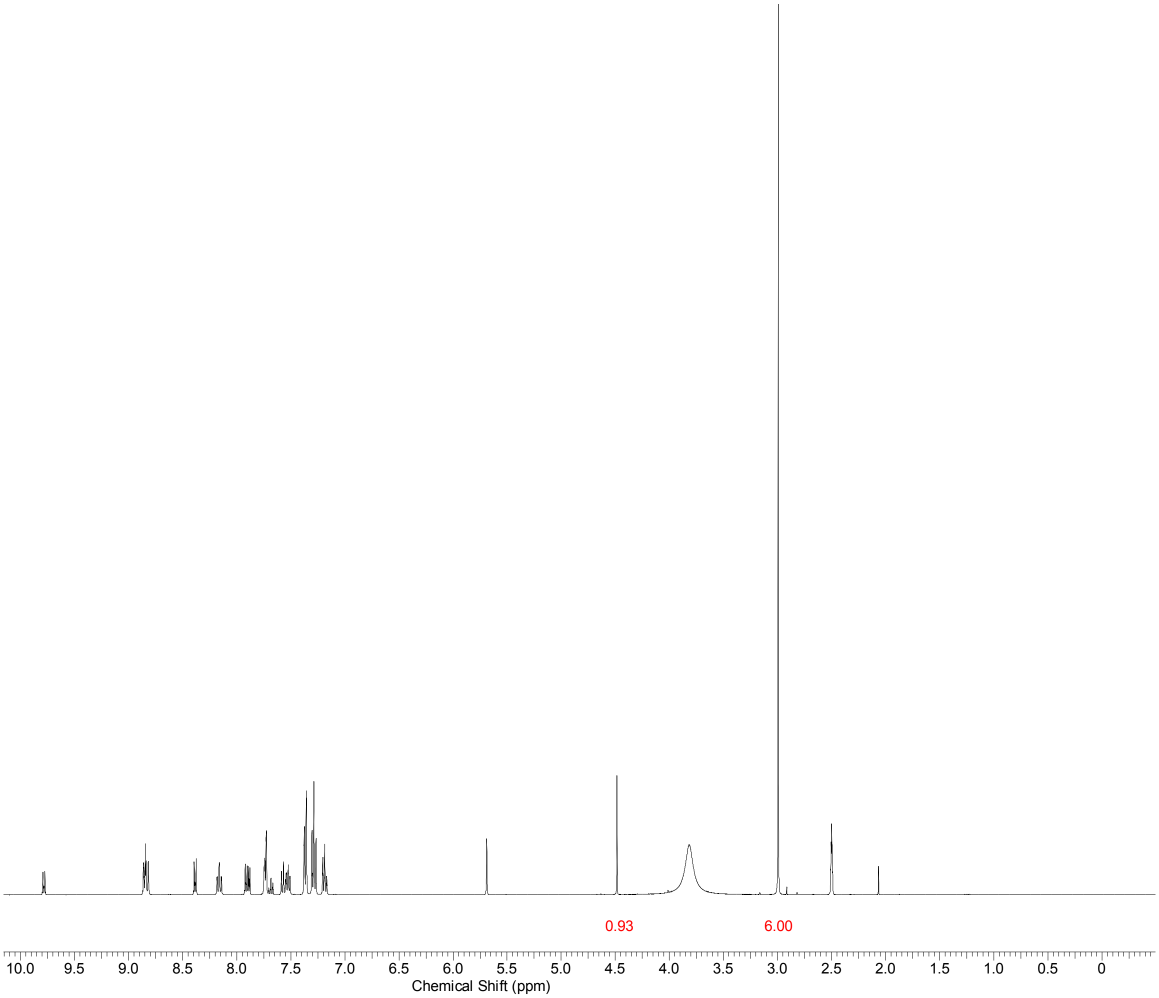

Figure S14: ^{1}H-NMR obtained after 6 min intermittent irradiation (t_{on} = 1 ms) [$Ru(bpy)_3Cl_2$ mediated oxidation of benzhydrol to benzophenone]

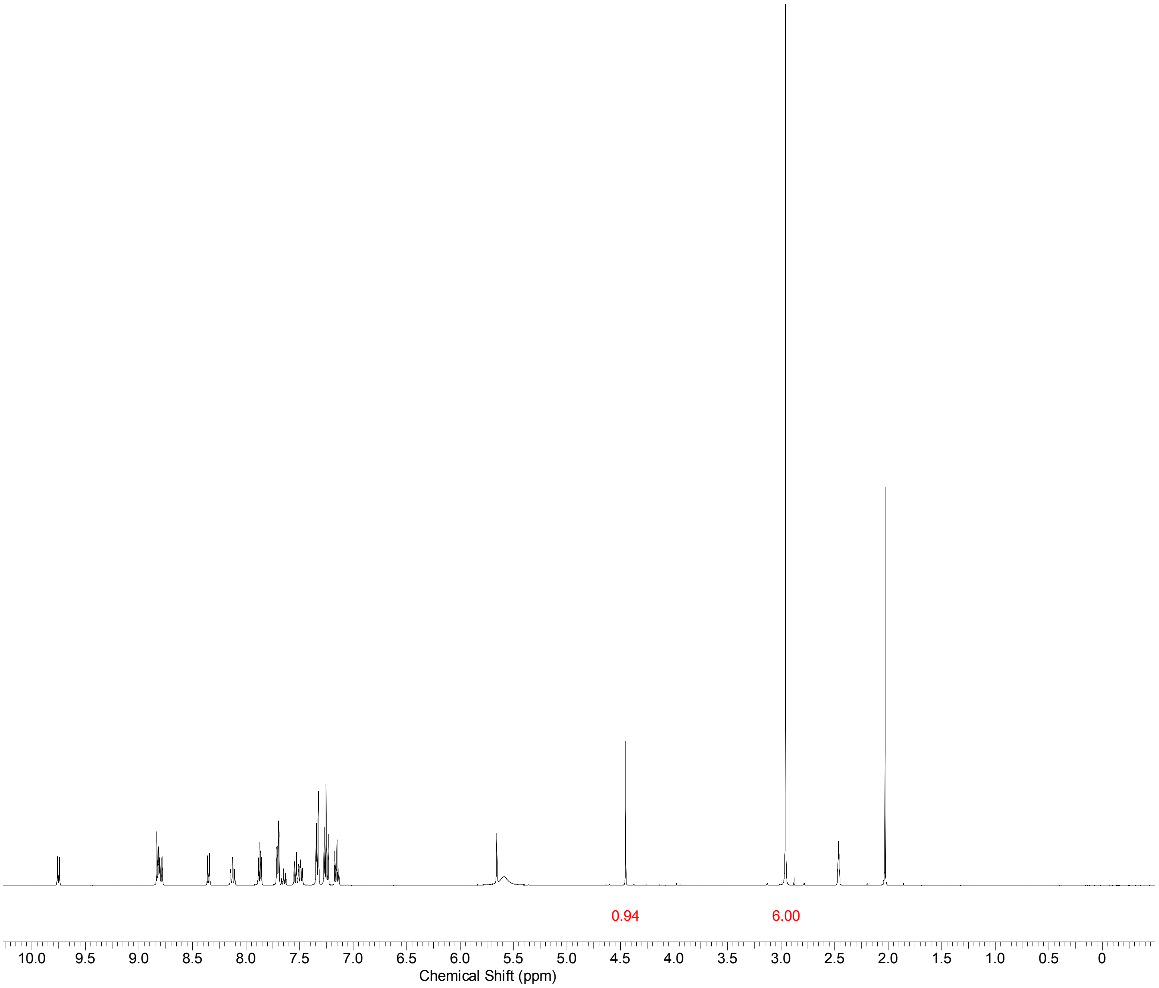

Figure S15: ^{1}H-NMR obtained after 6 min intermittent irradiation (t_{on} = 5 ms) [Ru(bpy)$_3$Cl$_2$ mediated oxidation of benzhydrol to benzophenone]

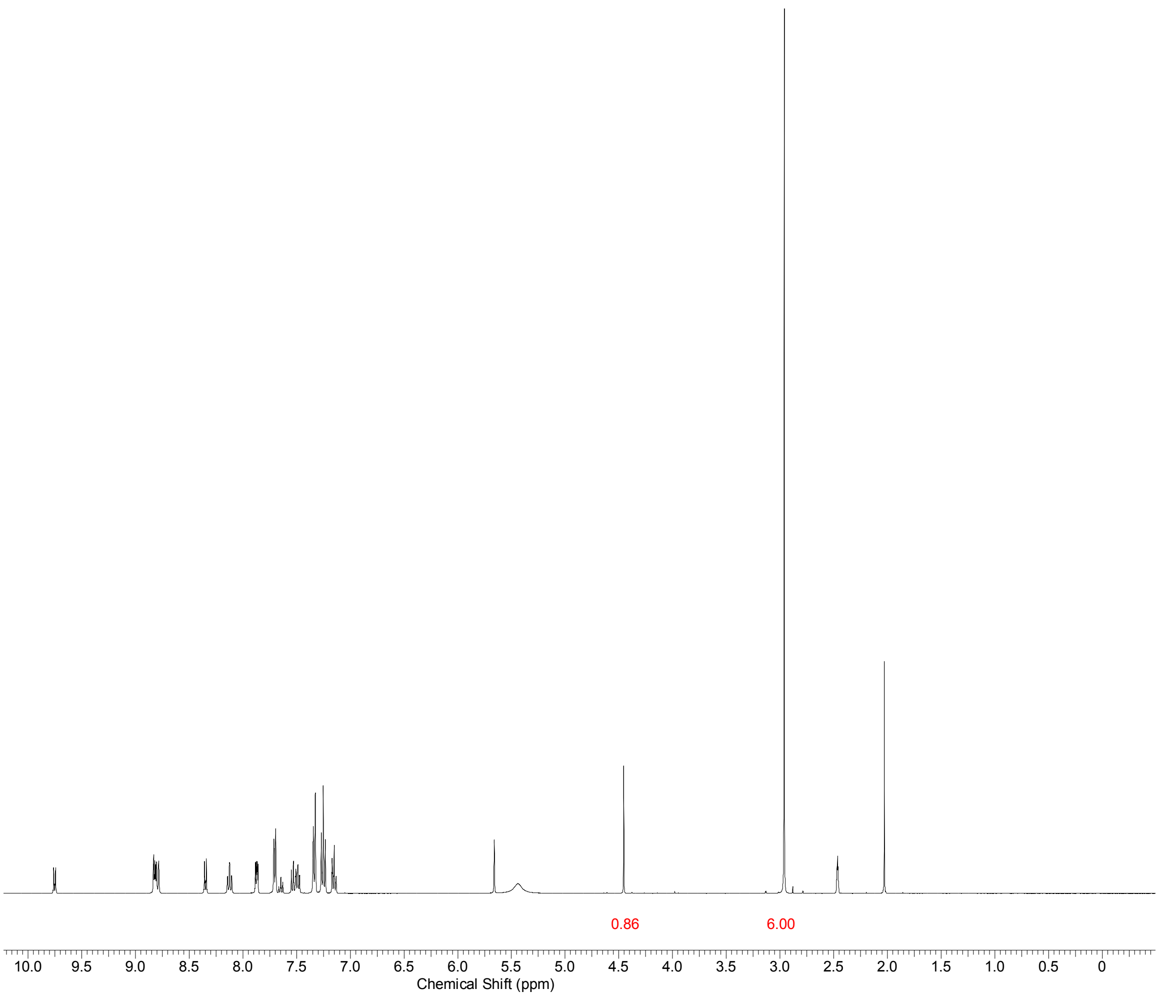

Figure S16: ^{1}H-NMR obtained after 6 min intermittent irradiation (t_{on} = 10 ms) [$Ru(bpy)_3Cl_2$ mediated oxidation of benzhydrol to benzophenone]

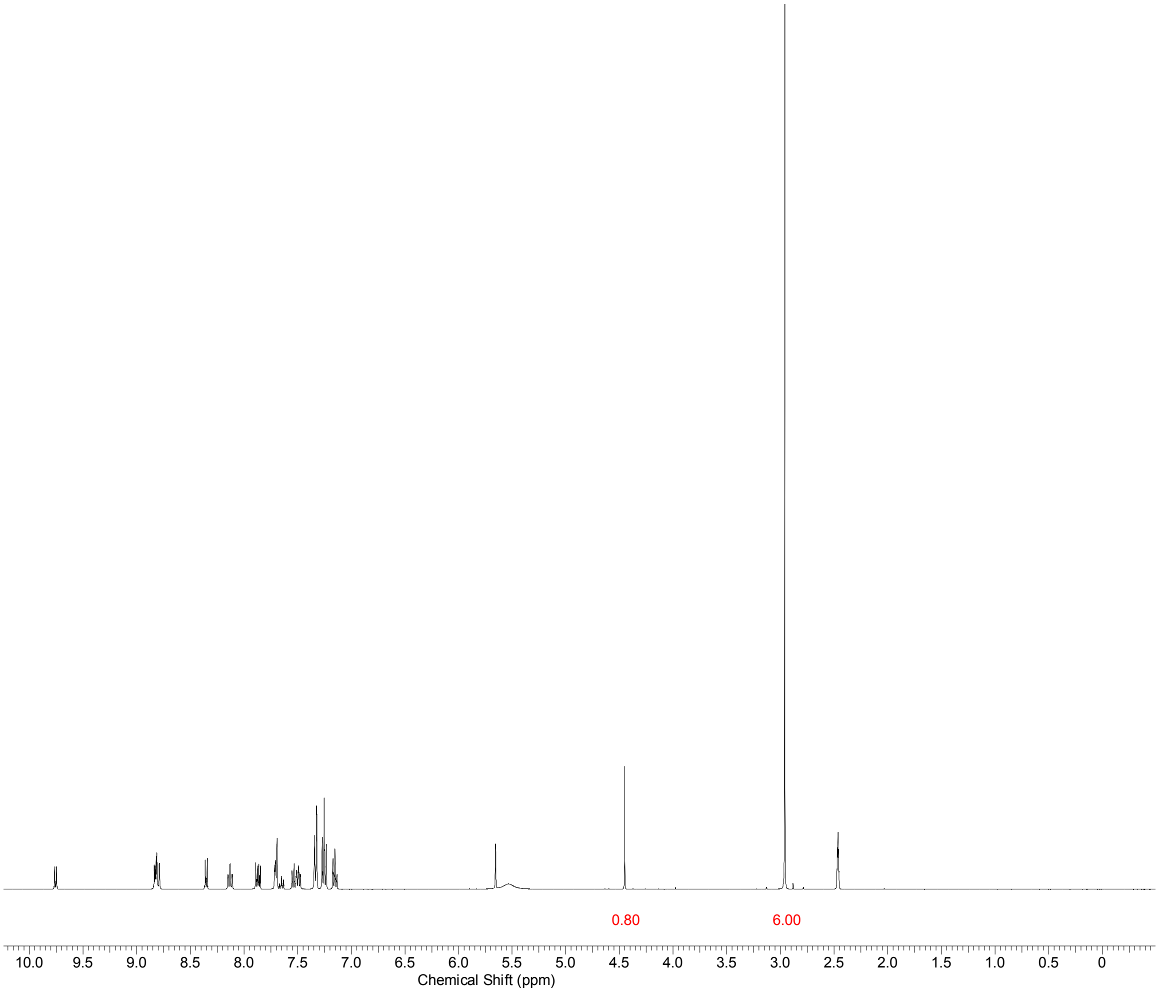

Figure S17: ^{1}H-NMR obtained after 6 min intermittent irradiation (t_{on} = 20 ms) [Ru(bpy)$_3$Cl$_2$ mediated oxidation of benzhydrol to benzophenone]

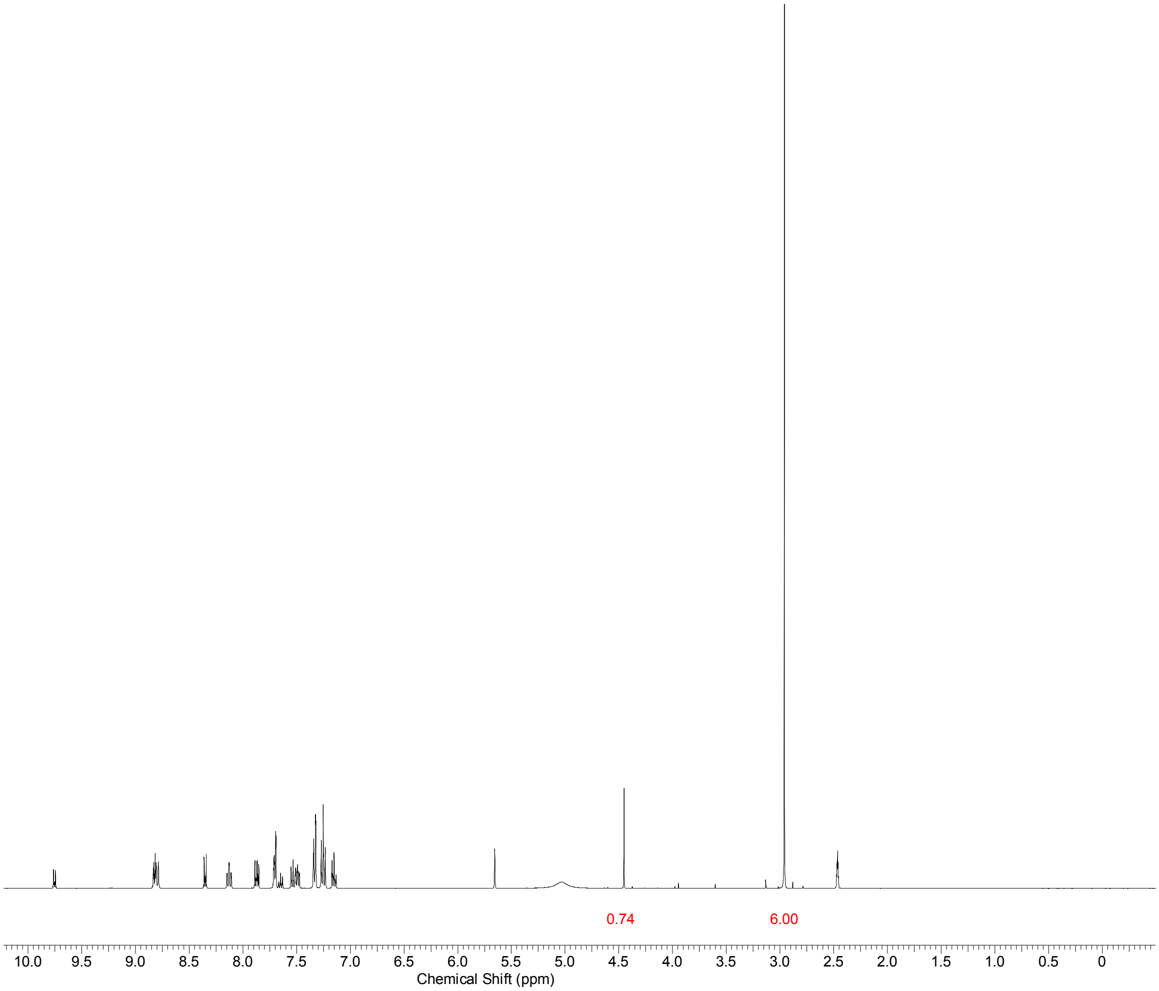

Figure S18: ^{1}H-NMR obtained after 6 min intermittent irradiation (t_{on} = 40 ms) [$Ru(bpy)_3Cl_2$ mediated oxidation of benzhydrol to benzophenone]

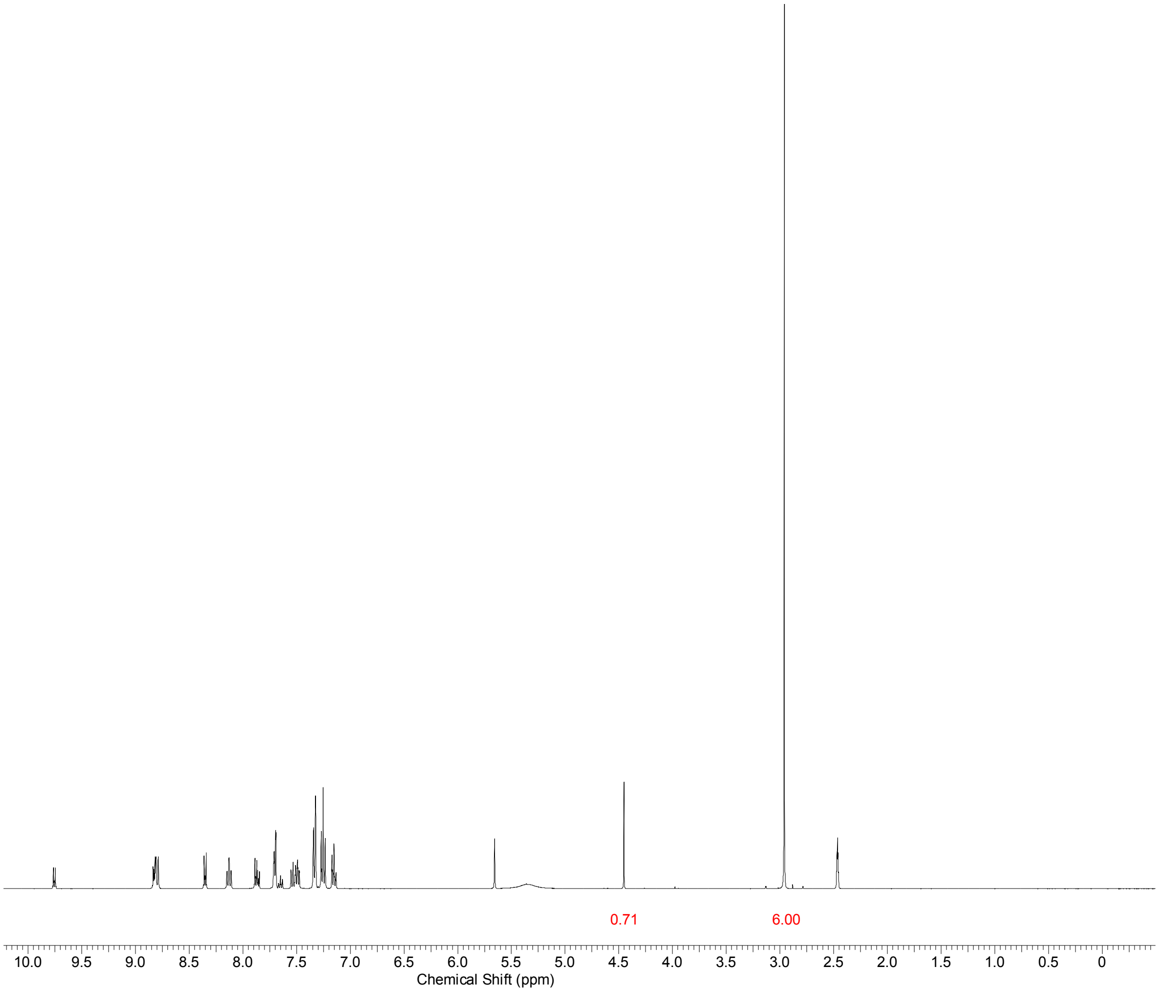

Figure S19: ^{1}H-NMR obtained after 6 min intermittent irradiation (t_{on} = 80 ms) [$Ru(bpy)_3Cl_2$ mediated oxidation of benzhydrol to benzophenone]

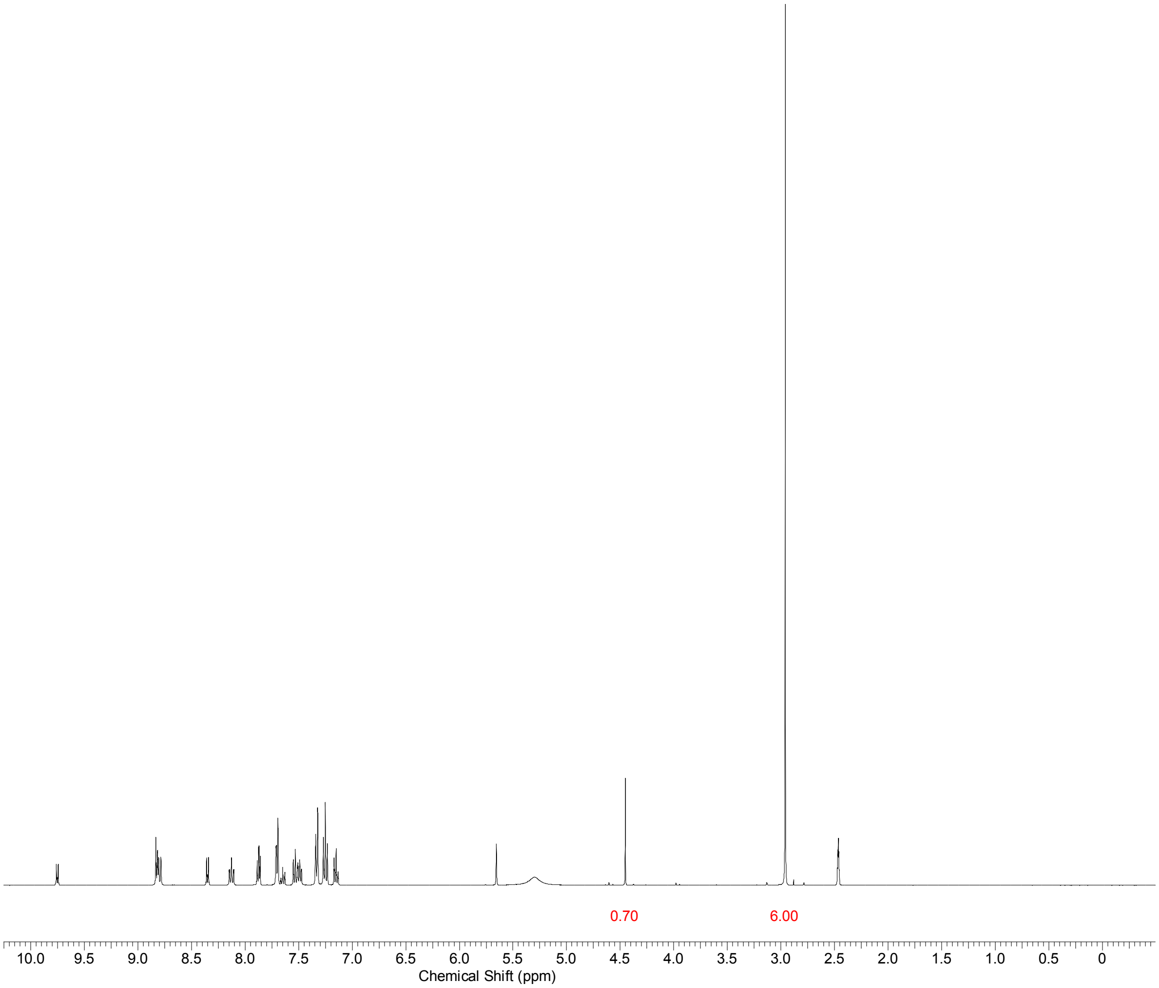

Figure S20: ^{1}H-NMR obtained after 6 min intermittent irradiation (t_{on} = 160 ms) [Ru(bpy)$_3$Cl$_2$ mediated oxidation of benzhydrol to benzophenone]

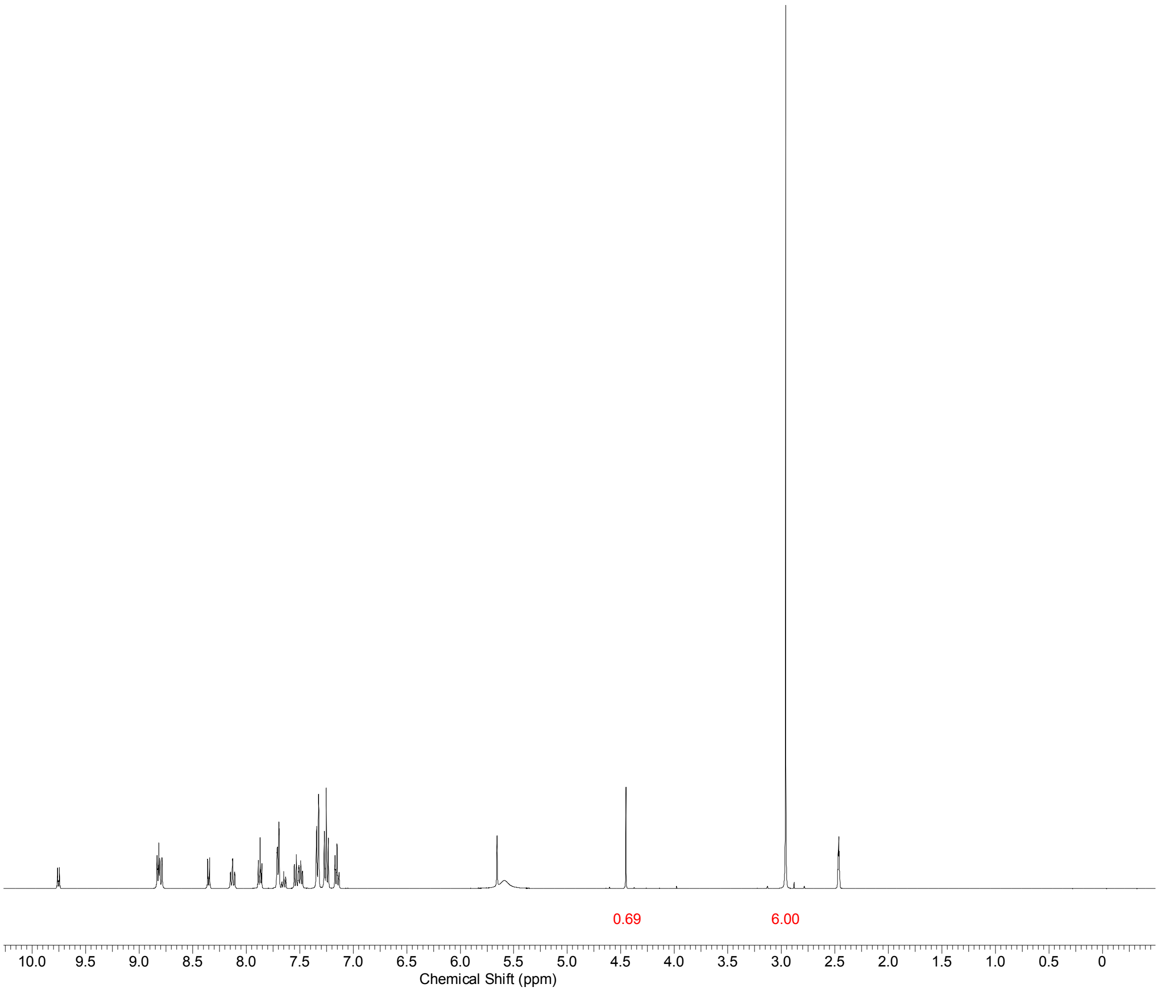

Figure S21: ^{1}H-NMR obtained after 6 min intermittent irradiation (t_{on} = 320 ms) [$Ru(bpy)_3Cl_2$ mediated oxidation of benzhydrol to benzophenone]

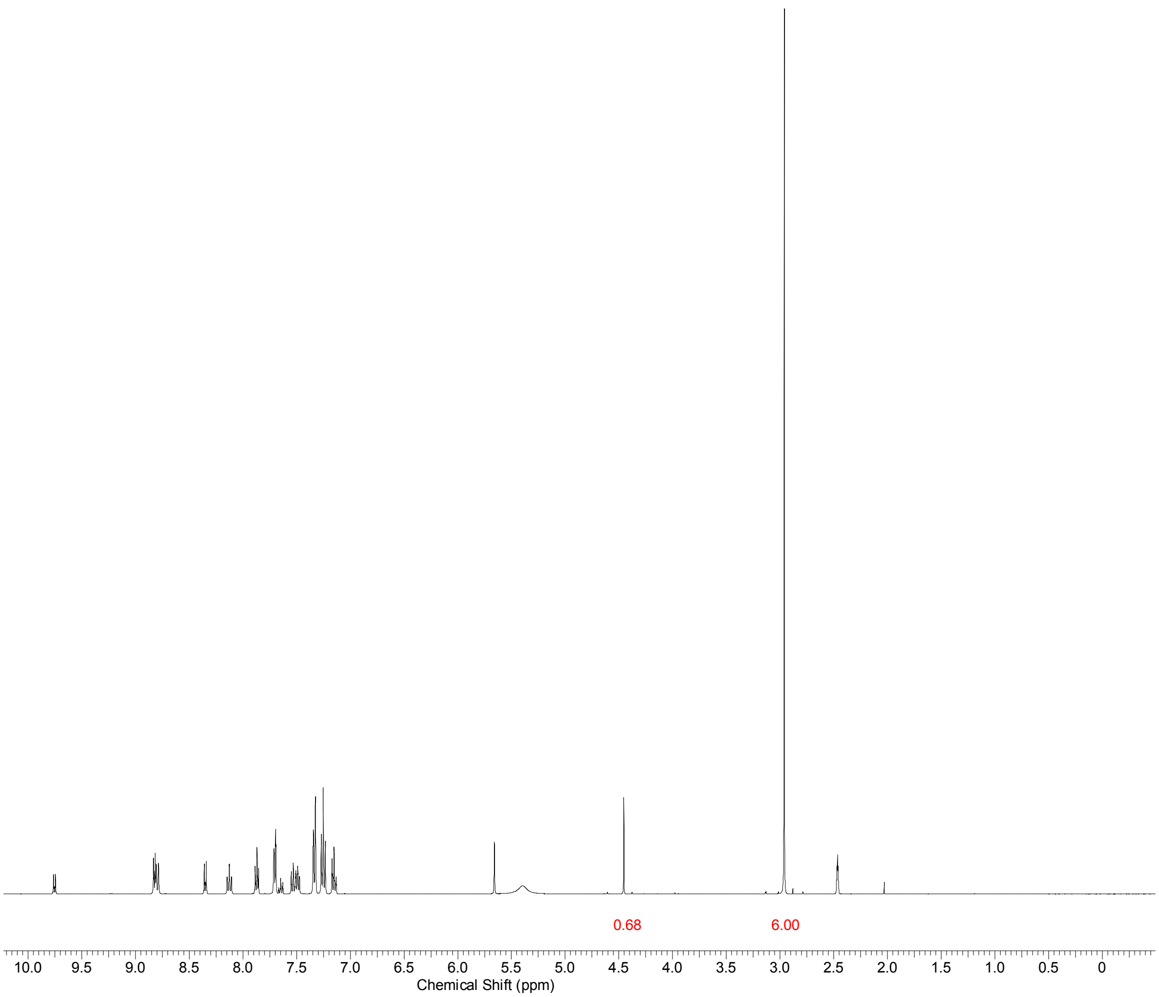

Figure S22: ^{1}H-NMR obtained after 6 min intermittent irradiation (t_{on} = 640 ms) [Ru(bpy)$_3$Cl$_2$ mediated oxidation of benzhydrol to benzophenone]

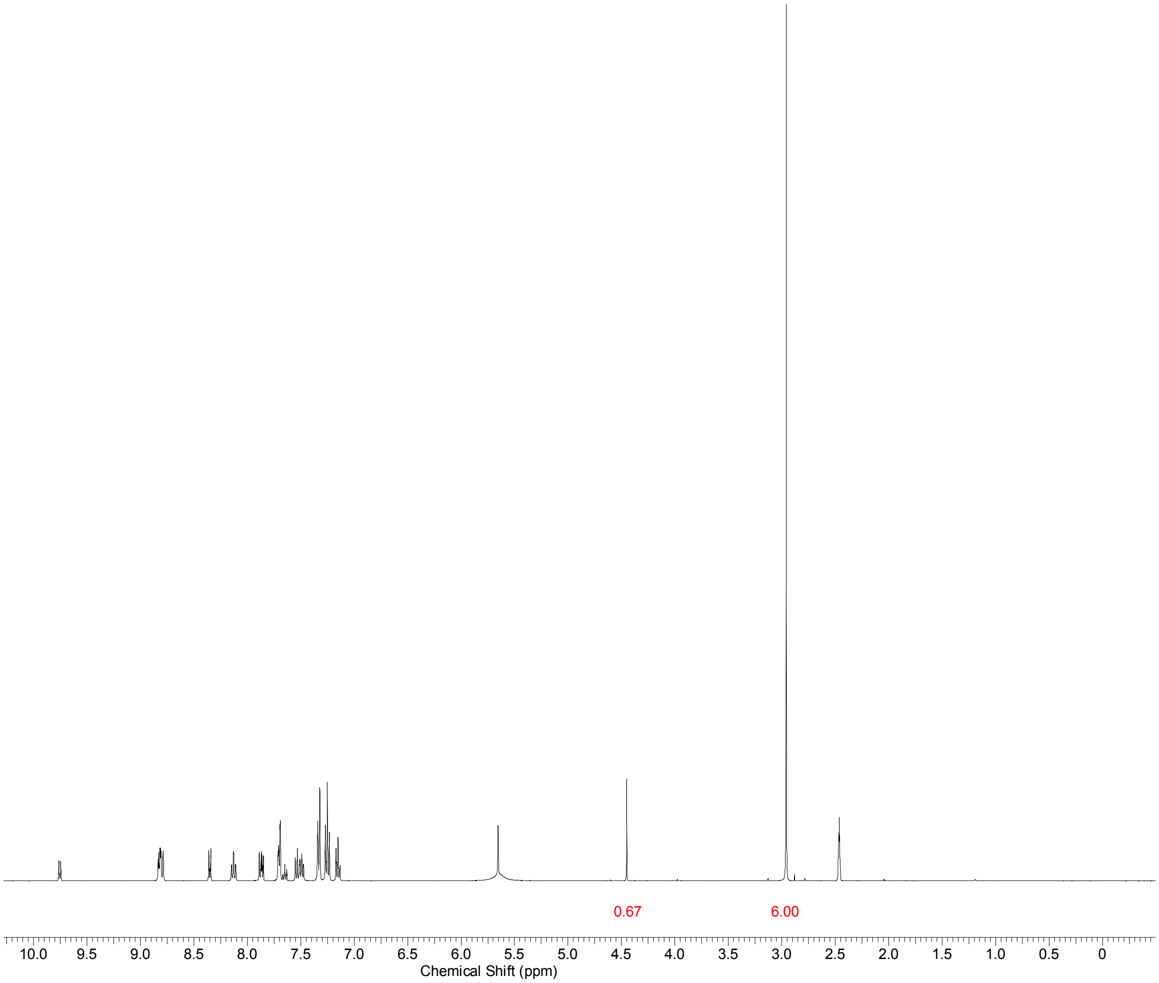

Figure S23: ^{1}H-NMR obtained after 6 min intermittent irradiation (t_{on} = 1280 ms) [$Ru(bpy)_3Cl_2$ mediated oxidation of benzhydrol to benzophenone]

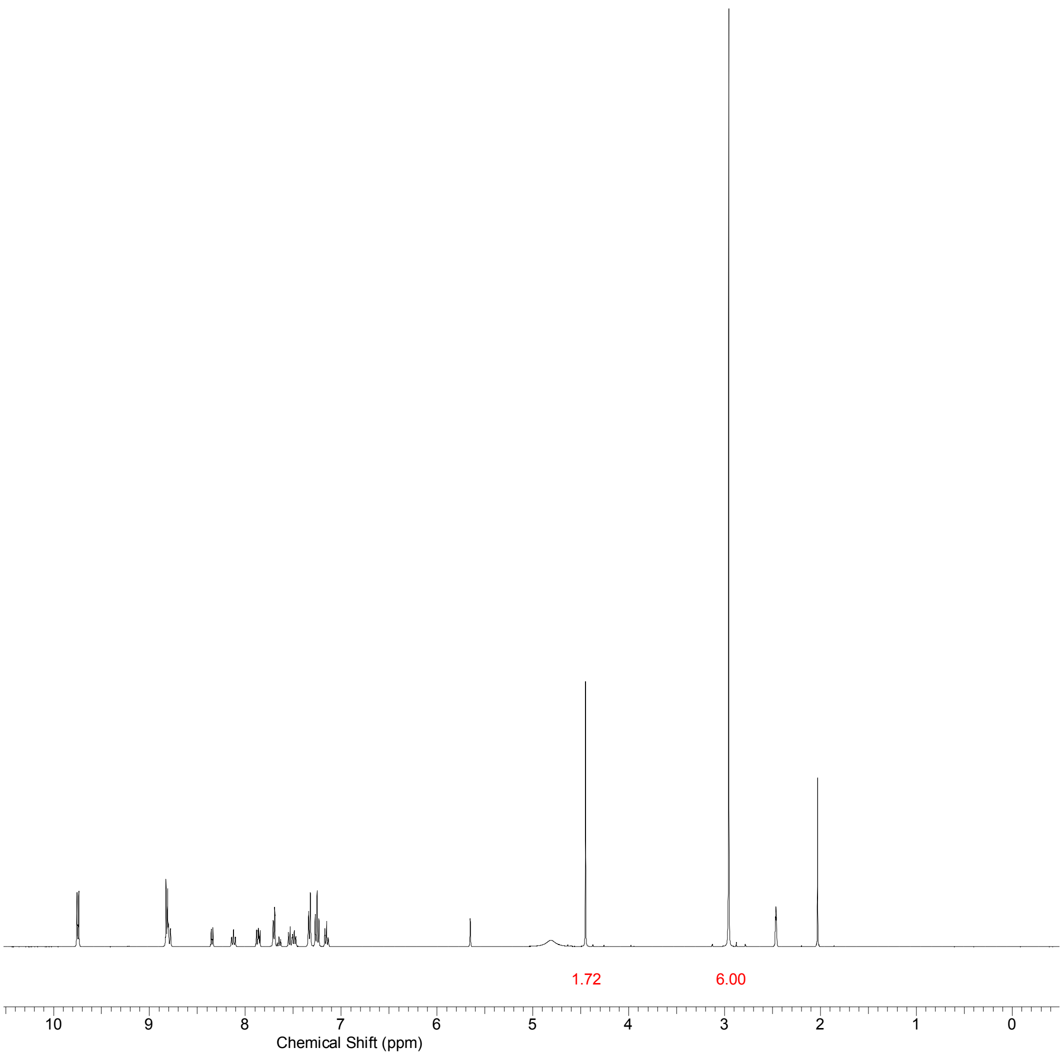

Figure S24: ^{1}H-NMR obtained after 2 min steady stare irradiation [Ru(bpy)$_3$Cl$_2$ mediated oxidation of benzhydrol to benzophenone] with 2X concentration of 4-cyano-*N*-methoxy pyridinium tetrafluoroborate

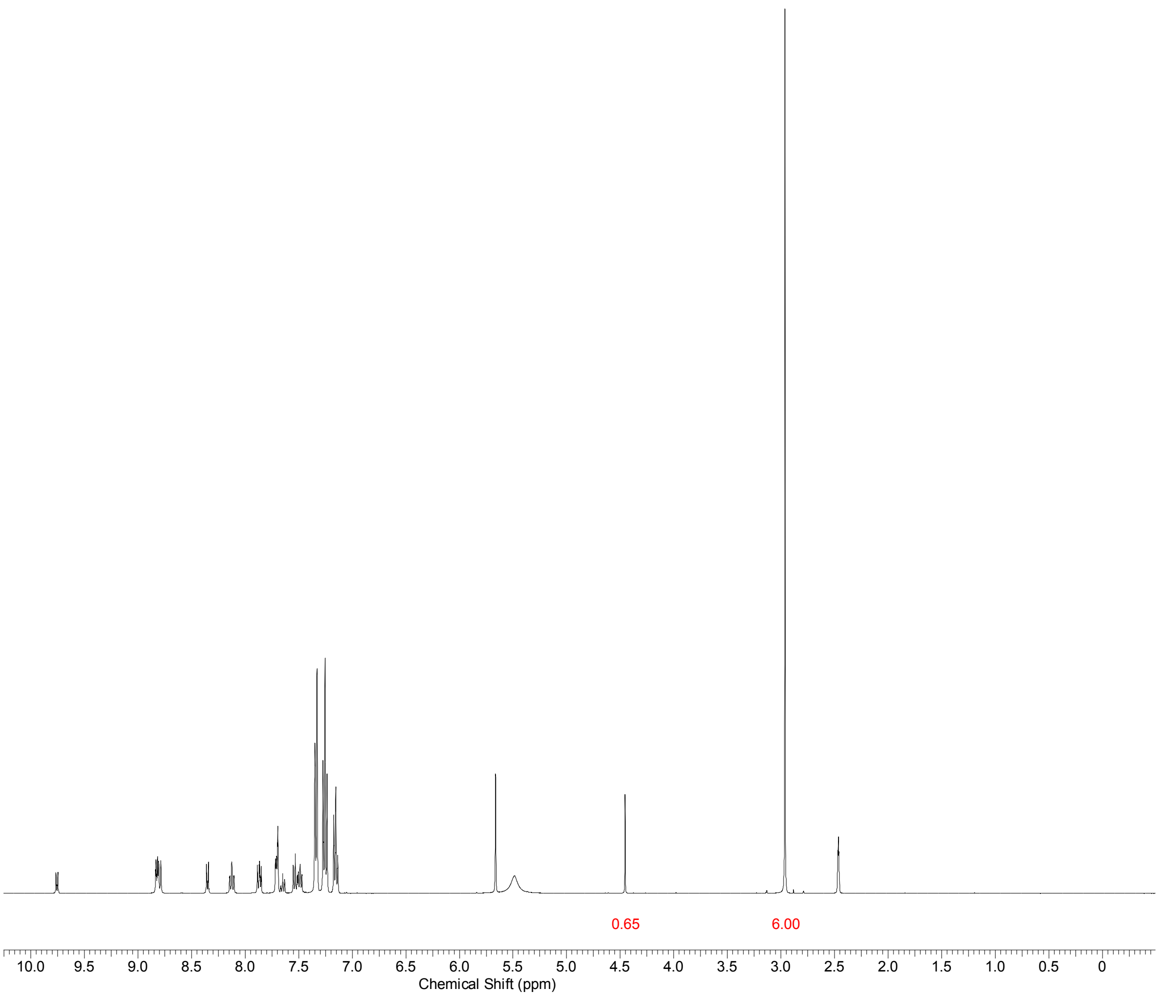

Figure S25: ^{1}H-NMR obtained after 2 min steady stare irradiation [Ru(bpy)$_3$Cl$_2$ mediated oxidation of benzhydrol to benzophenone] with 2X concentration of benzhydrol

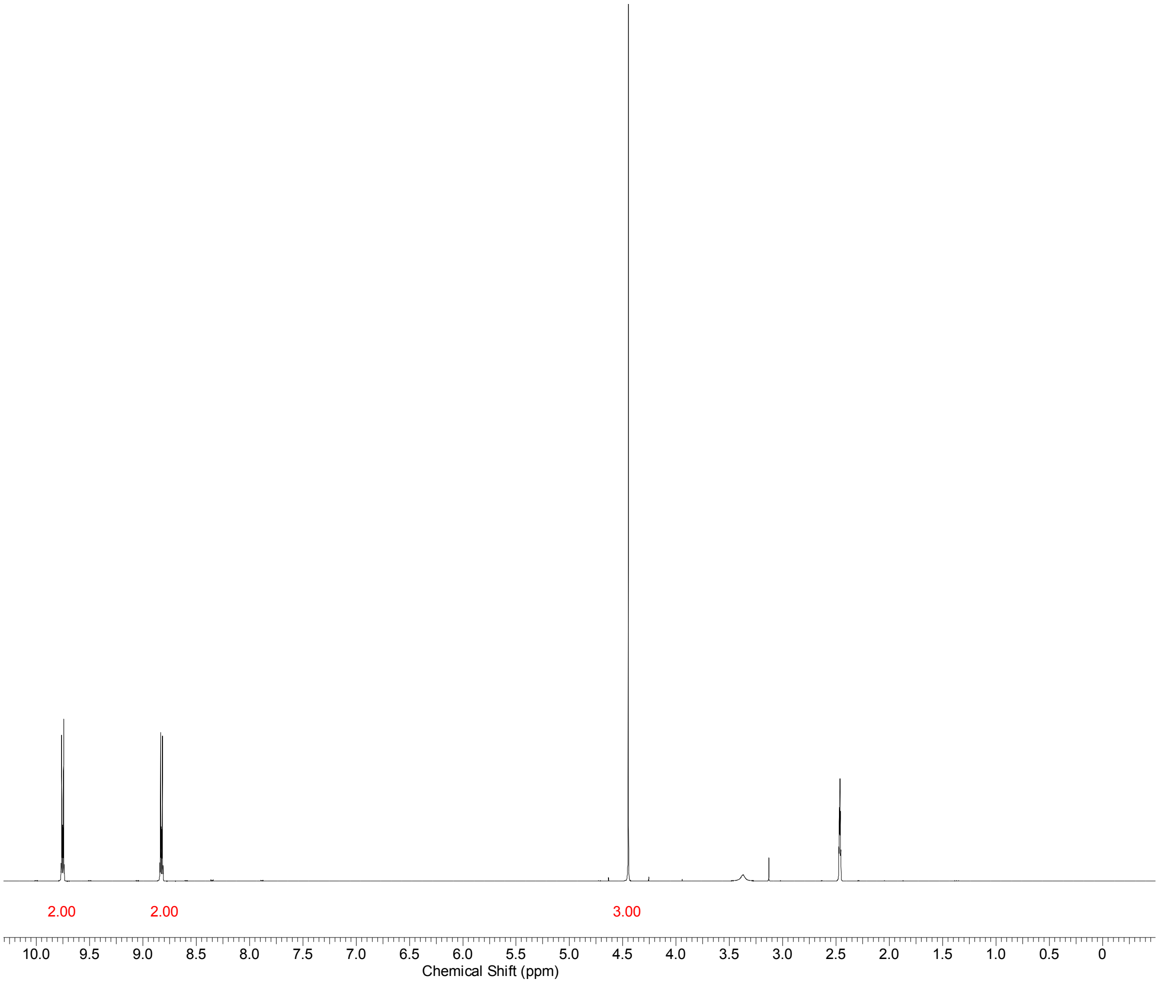

Figure S26: ^{1}H-NMR of 4-cyano-*N*-methoxy pyridinium tetrafluoroborate in DMSO-d6.

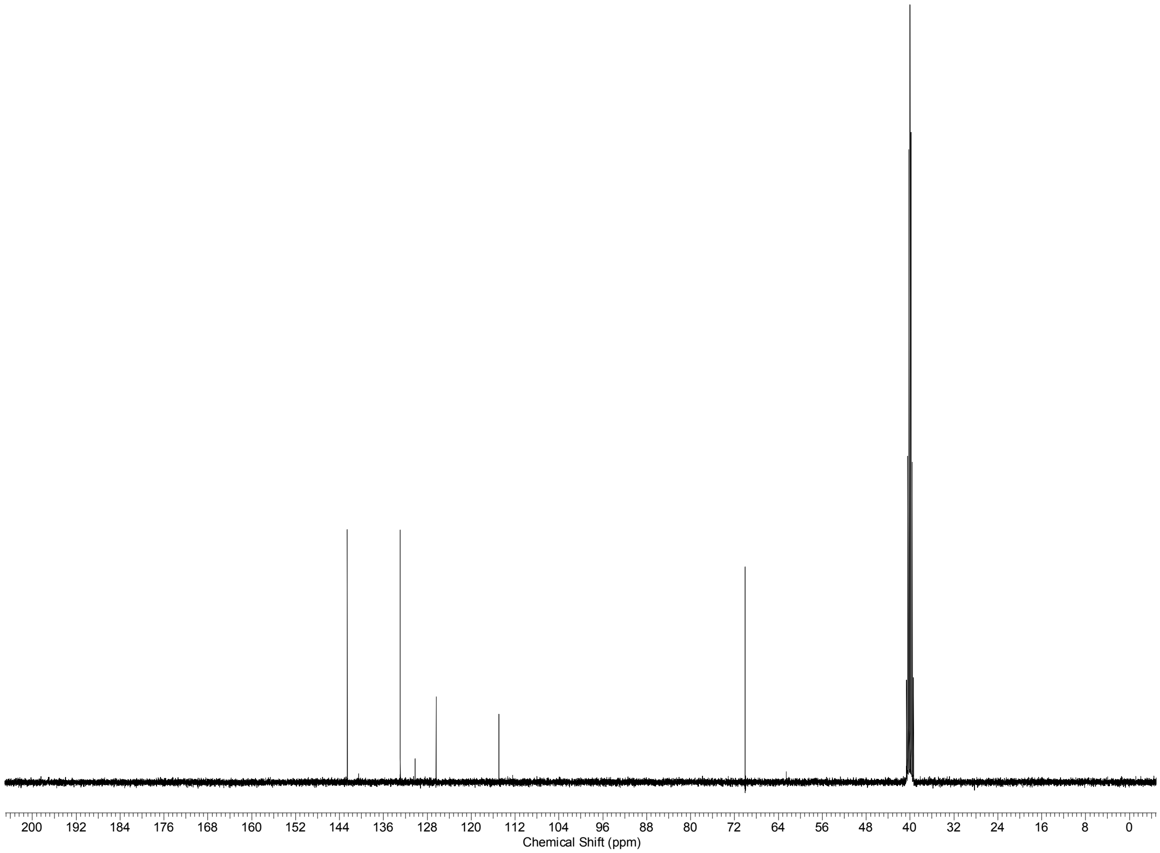

Figure S27: ^{13}C-NMR of 4-cyano-*N*-methoxy pyridinium tetrafluoroborate in DMSO-d6.

R. References

(1) Briers, F., Chapman, D. L.; Walters, E. LXXVII.-The influence of the intensity of illumination on the velocity of photochemical changes. The determination of the mean life of a hypothetical catalyst. *J. Chem. Soc.* **129**, 562-569,(1926).

(2) Burnett, G. M. & Melville, H. W. Determination of the Velocity Coefficients for Polymerization Processes. I. The Direct Photopolymerization of Vinyl Acetate. *Proc. R. Soc. London, Ser. A* **189**, 456-480,(1947).

(3) Ingold, K. U. Kinetic and mechanistic studies of free radical reactions in the 21st century. *Pure & Appl. Chem.* **69**, 241-243,(1997)

(4) Bovey, F. A. *Macromolecules: an introduction to polymer science.* 1-40 (Academic Press, INC., 1979).

(5) Weinberg, N.L., Weinberg, H.R. Electrochemical oxidation of organic compounds*Chem. Rev.* **68**, 449–523,(1968).
